# Pyrokinin receptor silencing in females of the southern cattle tick *Rhipicephalus* (*Boophilus*) *microplus* is associated with a reproductive fitness cost

**DOI:** 10.1186/s13071-022-05349-w

**Published:** 2022-07-11

**Authors:** Juan P. Wulff, Kevin B. Temeyer, Jason P. Tidwell, Kristie G. Schlechte, Caixing Xiong, Kimberly H. Lohmeyer, Patricia V. Pietrantonio

**Affiliations:** 1 Department of Entomology, Texas A&M University, College Station, TX 77843-2475 USA; 2 Knipling-Bushland U.S. Livestock Insects Research Laboratory and Veterinary Pest Genomics Center, United States Department of Agriculture-Agricultural Research Service (USDA-ARS), 2700 Fredericksburg Road, Kerrville, TX 78028-9184 USA; 3 Cattle Fever Tick Research Laboratory, USDA-ARS, 22675 N. Moorefield Rd. Building 6419, Edinburg, TX 78541-5033 USA

**Keywords:** Pyrokinin/pheromone biosynthesis-activating neuropeptide/diapause hormone (PK/PBAN/DH) neuropeptide family, Tick *CAPA* gene, GPCR, RNA interference, Tick survival, Tick fitness, Tick reproduction, Tick feeding

## Abstract

**Background:**

*Rhipicephalus* *microplus* is the vector of deadly cattle pathogens, especially *Babesia* spp., for which a recombinant vaccine is not available. Therefore, disease control depends on tick vector control. However, *R.* *microplus* populations worldwide have developed resistance to available acaricides, prompting the search for novel acaricide targets. G protein-coupled receptors (GPCRs) are involved in the regulation of many physiological processes and have been suggested as druggable targets for the control of arthropod vectors. Arthropod-specific signaling systems of small neuropeptides are being investigated for this purpose. The pyrokinin receptor (PKR) is a GPCR previously characterized in ticks. Myotropic activity of pyrokinins in feeding-related tissues of *Rhipicephalus* *sanguineus* and *Ixodes* *scapularis* was recently reported.

**Methods:**

The *R.* *microplus* pyrokinin receptor (*Rhimi-PKR*) was silenced through RNA interference (RNAi) in female ticks. To optimize RNAi, a dual-luciferase assay was applied to determine the silencing efficiency of two *Rhimi-PKR* double-stranded RNAs (dsRNA) prior to injecting dsRNA in ticks to be placed on cattle. Phenotypic variables of female ticks obtained at the endpoint of the RNAi experiment were compared to those of control female ticks (non-injected and beta-lactamase dsRNA-injected). *Rhimi-PKR* silencing was verified by quantitative reverse-transcriptase PCR in whole females and dissected tissues.

**Results:**

The *Rhimi-PKR* transcript was expressed in all developmental stages. *Rhimi-PKR* silencing was confirmed in whole ticks 4 days after injection, and in the tick carcass, ovary and synganglion 6 days after injection. *Rhimi-PKR* silencing was associated with an increased mortality and decreased weight of both surviving females and egg masses (*P* < 0.05). Delays in repletion, pre-oviposition and incubation periods were observed (*P* < 0.05).

**Conclusions:**

*Rhimi-PKR* silencing negatively affected female reproductive fitness. The PKR appears to be directly or indirectly associated with the regulation of female feeding and/or reproductive output in *R.* *microplus*. Antagonists of the pyrokinin signaling system could be explored for tick control.

**Graphical abstract:**

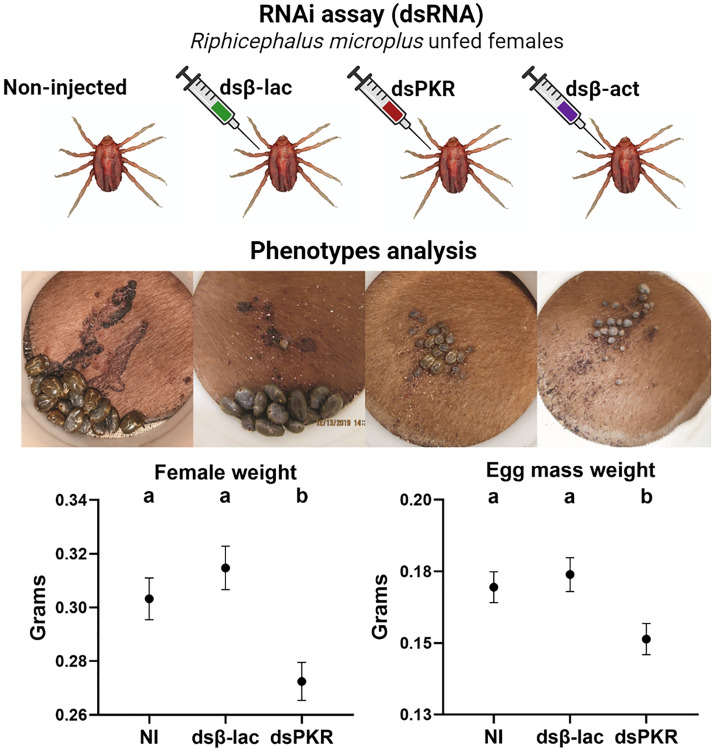

**Supplementary Information:**

The online version contains supplementary material available at 10.1186/s13071-022-05349-w.

## Background

In arthropods, neuropeptides and their receptors play a critical integrative regulatory role in many physiological processes, such as feeding, excretion, mating, molting and diapause [1, 2]. Although neuropeptides are likely as important in ticks (Acari: Ixodidae), little is known about their physiological functions in comparison to other groups such as insects and crustaceans [1–3]. The recognized significance of emerging tick-transmitted pathogens to humans, cattle and wildlife led to the genome sequencing of the Lyme disease vector, *Ixodes* *scapularis* Say (Acari: Ixodidae) [4] and the cattle tick *Rhipicephalus* (*Boophilus*) *microplus* Canestrini (Acari: Ixodidae) [5, 6]. In addition, *R.* *microplus* ticks have developed resistance to many acaricide classes and, thus, endocrinological research is crucial, specifically the study of neuropeptides and their G protein-coupled receptors (GPCRs), for the discovery of novel selective targets against ticks [1].

The Capability/Pyrokinin (*CAPA/PK*) endocrine signaling system is an ancient pathway in invertebrates, present in Nematoda and Arthropoda, that is putatively homologous of the Neuromedin U system in Vertebrata [7]. The ancestral neuropeptide *CAPA*/*PK* gene was duplicated and differentiated in insects into two genes, *CAPA*, which encodes periviscerokinins, and pyrokinin/pheromone biosynthesis-activating neuropeptide (*PK*/*PBAN*) [8].

The *PK*/*PBAN* gene encodes the pyrokinin/pheromone biosynthesis-activating neuropeptide/diapause hormone (PK/PBAN/DH) neuropeptide family. This family is defined by a conserved 5-amino acid C-terminal sequence (FXPRL-amide, X = G, T, I, V, K, A, P, S or D), which is the minimal sequence required for physiological activity [9]. This motif is conserved throughout several arthropod taxa, such as Arachnida [10–12], Protura [13], Crustacea [3, 14, 15] and Insecta (reviewed in [9]). Peptides of the PK/PBAN/DH family are myotropic and promote pheromone biosynthesis in different insect orders [9]. In addition, they regulate other physiological process in Lepidoptera, such as melanization [16], induction of embryonic diapause and termination of pupal diapause [17, 18], puparium formation [19] and ecdysteroidogenesis in prothoracic glands [20].

*PK/PBAN/DH* orthologous sequences from insects, and their receptors, have been identified in ticks [4, 10, 21–23]. To date, only two functional studies with tick PK recombinant receptors of *R.* *microplus* [23] and *I.* *scapularis* [24] have been conducted, and only recently we reported the first in vitro analysis of tick pyrokinin 2 myotropic activity on *I.* *scapularis* and *Rhipicephalus* *sanguineus* tissues [25].

The pyrokinin receptor (PKR) is a GPCR, characterized by seven transmembrane helical regions, belonging to GPCR family A. The corresponding locus has been annotated in *I.* *scapularis* as pyrokinin-1 receptor LOC8040758. Three receptor isoforms corresponding to three transcripts (X1, XP_042145382.1; X2, XP_002401180.3; and X3, XP_029839423.1, respectively) have been predicted for this locus [4]. These three isoforms share a 94% identity in the overlapping amino acid sequences of the coding regions, and the X1 and X2 variants are longer than the X3 variant (by 83 and 80 amino acid residues, respectively) at the C-terminal region [4]. The pyrokinin-like receptor protein predicted in *Dermacentor* *variabilis* (Say) (Acari: Ixodidae) (ACC99623.1, unpublished) corresponds to the X1 variant predicted in *I.* *scapularis,* and the pyrokinin-like receptor protein predicted in *Dermacentor* *silvarum* Olenev (Acari: Ixodidae) (XP_037556591.1, unpublished) corresponds to the X3 variant of *I.* *scapularis*. Experimental confirmation only exists in *R.* *microplus* [23] and *I.* *scapularis* [24] by cloning of complementary DNAs (cDNAs) that correspond to the *I.* *scapularis* X3 variant. This is the only form identified in *R.* *microplus* by both analysis of the genomic sequence and by cDNA cloning, and is the focus of the present work. The genomic analyses of *R.* *microplus* and other tick species predicted the PKR as similar to the *I.* *scapularis* X3 variant [5, 6]. The transcript relative expression pattern in both tick species showed that the highest expression for PKR was in the synganglion, and secondly in the female reproductive system [23, 24]. However, a lower expression was observed in hindgut, and a very low expression was observed in other tissues, such as midgut, Malpighian tubules and salivary glands [23, 24]. This expression pattern suggests a functional role in feeding and reproduction, possibly associated with the regulation of myotropic activity, as we recently reported [25].

Beyond a few neuropeptides and cognate GPCRs characterized in ticks [10, 23, 26–29], little is known about the physiological role of GPCRs in this taxa, but some have shown a potential for interventions after RNA interference (RNAi) experiments by decreasing tick fitness [28]. Subsequently, high-throughput screens were conducted with the aim to discover new chemistries of antagonists of the tick kinin receptor [30]. The significance of these approaches is underscored by the lack of recombinant vaccines to prevent deadly babesiosis of cattle caused by *Babesia* spp. which is transmitted by *R.* *microplus*, limiting this disease control to tick control [31, 32]. Further, *R.* *microplus* populations worldwide have developed resistance to amidines (amitraz), organophosphates, pyrethroids, fluazuron and ivermectins (reviewed in [33–37]). Therefore, there is a critical need to validate novel and selective pesticide targets in ticks to ensure the global security of cattle herds. To this end and to help elucidate the function of the tick pyrokinin system, we investigated the PKR loss of function through gene silencing in females of *R.* *microplus.*

## Methods

### Tick rearing and animal care

*Rhipicephalus* *microplus* ticks were reared at the Cattle Fever Tick Research Laboratory (USDA-ARS; Mission, TX, USA) under a cooperative agreement with Texas A&M AgriLife Research. Cattle breeds used for tick production or gene silencing experiments were *R.* *microplus*-naïve Hereford, Charolais or Angus, with each individual weighing between 136 and 182 kg. All cattle were vaccinated, dewormed and acclimated for 2 weeks at the USDA-ARS Knipling-Bushland U.S. Livestock Insects Research Laboratory prior to shipment to the USDA-ARS Cattle Fever Tick Research Laboratory (CFTRL), a biosecure research facility near Edinburg, Texas, USA. Cattle used for routine tick rearing or RNAi experiments at the CFTRL were maintained under approved Animal Use Protocols (AUP). All procedures for handling and treating animals were approved by the Texas A&M University Institutional Animal Care and Use Committee (IACUC), USA, TAMU AUP 2019-0197 EX, referring to IACUC USDA-ARS approved AUP 2021-12. Ticks used in this study were obtained from colonies of the *R.* *microplus* acaricide-susceptible Deutsch strain established from ticks collected in 2001 from an outbreak in Webb County, Texas, USA [38]. For the RNAi experiments, filial generation F65 was used for RNAi tests performed on December 2019; F66 for the March 2020 RNAi replicate; and F80 for the July 2021 RNAi replication.

### RNA isolation, cDNA synthesis and quantitative reverse-transcriptase PCR analysis

Whole ticks and dissected tissues (synganglion, ovary and tick carcasses) were used for molecular analyses. For analyses of expression in different stages we used adults of both sexes, nymphs, neolarvae and eggs. Prior to RNA extraction, whole ticks and tissues were placed in Trizol reagent™ and disrupted using the Bead Mill Homogenizer Omni Bead Ruptor 12 (Omni International, Inc., Waterbury, CT, USA) with an equal proportion of 1.4- and 2.8-mm ceramic beads for 1 min (ovary and synganglion) or 3 min (for whole ticks adults, nymphs, larvae, egg masses and carcasses), at 5.65 Hz (m/s). Total RNA was extracted from whole ticks and individual tick tissues using the Zymo Quick-RNA™ Microprep kit (Zymo Research, Irvine, CA, USA), according to the manufacturer's specifications. Two DNAse 1 (deoxyribonuclease 1) steps were conducted: (i) during RNA extraction, by adding 30 U of DNAse 1 to the sample and incubating for 15 min at room temperature (RT); and (ii) after solubilization of the sample in nuclease-free water (NF-water), by adding 5 U of DNAse 1 and incubating for 15 min at RT. After DNAse 1 treatment, the RNA Clean & Concentrator™-5 Kit (Zymo Research) was used to clean the sample (following the manufacturer's specifications), and the sample was recovered in 13 µl of NF-water. Clean total RNA (2 µl) was quantified spectrophotometrically using a Tecan Infinite M200 Pro plate reader (Tecan, Research Triangle Park, NC, USA).

Total RNA from whole ticks or tissues was used for cDNA synthesis in a final reaction volume of 22 μl containing 150–200 ng of total RNA as template (the same for all tissues), 1 µl oligo-dT20 (50 µM) and 1 µl random hexamers (50 ng/µl), using the SuperScript™ III First-Strand Synthesis System (Invitrogen™, Thermo Fisher Scientific, Waltham, MA, USA) according to the manufacturer's specifications. The synthesized cDNA concentration (2 µl of a dilution 1:10) was checked using a Tecan Infinite M200 Pro plate reader (Tecan), and the undiluted cDNA was stored at − 20 °C until further use.

Quantitative reverse-transcriptase PCR (qRT-PCR) analysis was performed in a reaction volume of 10 µl consisting of 5 µl PowerUp SYBR™ Green PCR Master Mix (Applied Biosystems™, Thermo Fisher Scientific), 1 µl of a primer mix (300 nM final concentration of each primer), 2 µl of cDNA (40 ng/µl) and 2 µl of NF-water. All reactions were performed in duplicate. Real-time relative quantification was performed using the QuantStudio 6 Flex Real-Time PCR System (Applied Biosystems, Thermo Fisher Scientific). The conditions for qRT-PCR cycling were: an initial denaturation step of 10 min at 95 °C, followed by 40 cycles of 95 °C 15 s and 60 °C 60 s. All oligonucleotide primers (Table 1) used for qRT-PCR analysis were synthesized by IDT Integrated DNA Technologies (Coralville, IA, USA).

**Table 1:** Oligonucleotide primers for cloning, double-stranded RNA synthesis and quantitative reverse-transcriptase PCR

Primer name	Oligo sequence 5ʹ-3′^a^	Notes
RmPyro93U27	TGGAACTGTCAAGCAGGCTGAGGCAGA	Dualluc construction
RmPYro1625L30	AGAGAGTAAGCTTTCGCAGGCAAAATACAC	Dualluc construction
DualLucRmPyr-8U18	CGTCAGCGGGCTTCGAACAAGCAGGCTGAGGCAGA	Dualluc construction (insert)
DualLucPyr-1538L21	GAATACTGTGGGAGCTCGCTA	Dualluc construction (insert)
DualLucPyro1789U21	GAATACTGTGGGAGCTCGCTA	Dualluc construction (vector)
DualLucPyro8198L16	TTCGAAGCCCGCTGAC	Dualluc construction (vector)
RmPyro-762U20	GCGAGAGGAGCCTCAACGAG	*Rhimi-PKR* dsRNA synthesis
RmPyro-913L20	GTACCGGTGTCGTCTTCGTC	*Rhimi-PKR* dsRNA synthesis
RmPyro-762U20T7	*AAAGGCCTTAATACGACTCACTATAGGG*GCGAGAGGAGCCTCAACGAG	*Rhimi-PKR* dsRNA synthesis
RmPyro-913L20T7	*AAAGGCCTTAATACGACTCACTATAGGG*TCGGTGTCGTCTTCGTC	*Rhimi-PKR* dsRNA synthesis
RmPyro-1485U16	TCGCCGCCAAGTACAG	*Rhimi-PKR* dsRNA synthesis
RmPyro-1627L27	GAGAGTAAGCTTTCGCAGGCAAAATAC	*Rhimi-PKR* dsRNA synthesis
RmPyro-1485U16-T7	*AAAGGCCTTAATACGACTCACTATAGGG*TCGCCGCCAAGTACAG	*Rhimi-PKR* dsRNA synthesis
RmPyro-1627L27-T7	*AAAGGCCTTAATACGACTCACTATAGGG*AGAGTAAGCTTTCCAGGCAAAATAC	*Rhimi-PKR* dsRNA synthesis
Amp-fwd	CGCTGGTGAAAGTAAAATATG	Beta-lactamase dsRNA synthesis [28]
Amp-rev	GCCGGGAAGCTAGAGTAAGTA	Beta-lactamase dsRNA synthesis [28]
Amp-T7	*TAATACGACTCACTATAGGG*CGCTGGTGAAAGTAAAATATG	Beta-lactamase dsRNA synthesis [28]
Amp-T7	*TAATACGACTCACTATAGGG*CCGGGAAGCTAGAGTAAGTA	Beta-lactamase dsRNA synthesis [28]
BmBActin-1U20	TCCTCGTCCCTGGAGAAGTC	*Rhimi-ACTB* dsRNA synthesis [28]
BmBActin-285L18	GGGGGAGCGATGATCTTG	*Rhimi-ACTB* dsRNA synthesis [28]
BmbActin-1U20-T7	*TAATACGACTCACTATAGGG*TCCTCGTCCCTGGAAGAAGTC	*Rhimi-ACTB* dsRNA synthesis [28]
BmbActin-285L18-T7	*TAATACGACTCACTATAGGG*GGAGCGATGATCTTG	*Rhimi-ACTB* dsRNA synthesis [28]
RmPKR-qF2	ACGCGCCATGAATGGAA	*Rhimi-PKR* qRT-PCR [23]
RmPKR-qR2	GTGTGAAGCTGGTGGTTTGAGA	*Rhimi-PKR* qRT-PCR [23]
BmbA-1528-F	CAAACGGAGGTGGAGCTGTC	*Rhimi-ACTB* qRT-PCR [28]
BmbA-1629-R	GCTAGAATATGTGAGGGCGCGAC	*Rhimi-ACTB* qRT-PCR [28]
BmELF1a-88-F	CGTCTACAAGATTGGTGGCATT	*Rhimi-EF1A* qRT-PCR [39]
BmELF1a-196-R	CTCAGTGGTCAGGTTGGCAG	*Rhimi-EF1A* qRT-PCR [39]
RmRPS4-qF1	TCATCCTGCACCGCATCA	*Rhimi-RPS4* qRT-PCR [23]
mRPS4-qR1	ACGCGGCACAGCTTGTACT	*Rhimi-RPS4* qRT-PCR [23]

*ACTB* Beta-actin gene, *ds* double-stranded,* EF1A* elongation factor 1 alpha gene, *PKR* pyrokinin receptor,* qRT-PCR* quantitative reverse-transcriptase PCR,* Rhimi* *Rhipicephalus microplus*, genus and species, *RPS4* ribosomal protein S4 gene

^a^Sequences in italics were added to the primers and are not part of the tick complementary DNA sequences

To select reference genes for qRT-PCR analyses, we tested their stability across different treatments by selecting samples randomly. Four genes which had been used previously in published studies on *R.* *microplus* [23, 28, 39] were tested for this purpose, namely alpha and beta tubulin (*Rhimi-A-Tub*, XM_037432254.1 and *Rhimi-B-Tub,* XM_037427816.1 respectively), elongation factor 1 alpha (*Rhimi-EF1A,* EW679365.1) and ribosomal protein S4 (*Rhimi-RPS4;* CV436347). The stability and suitability of these reference genes were evaluated with BestKeeper, Normfinder, Genorm and the comparative Delta-Ct method software tools [40]. *Rhimi-EF1A* and *Rhimi-RPS4* relative expression was the most stable and thus these two genes were selected as internal reference genes (Additional file 6: Table S1). The normalized relative quantity (NRQ) with respect to these reference genes was calculated for *Rhimi-PKR* and *R.* *microplus* beta-actin (*Rhimi-ACTB*) following the formulas in [41].

### *Rhimi-PKR* relative expression throughout different stages of development

To determine *Rhimi-PKR* relative expression by qRT-PCR across different stages of development, unfed neolarvae (early first larval instar [42]), nymphs and adults were flash frozen within 24 h of emergence and kept in 500 µl of RNAlater™ (Invitrogen) at − 80 °C until used for qRT-PCR analysis. All tick stages were maintained within two patches on a Hereford calf, with each patch infested with approximately 250 mg of tick neolarvae. To obtain newly molted nymphs, engorged larvae were removed from the host animal 6–7 days after neonate infestation. The larvae were allowed to molt in an environmental chamber kept at 25 ± 2 °C and a relative humidity (RH) of 95% [43]. Engorged nymphs were removed from the animal after 13 to 14 days post-infestation (dpi). These were placed in an environmental chamber maintained at the same temperature and RH as stated above, until ecdysis. Eight biological replicates (*n* = 8) for each of the developmental stages were used, as follows: for egg masses, each replicate consisted of approximately 100 mg of eggs that were a pool from egg masses from different females (approx. 11.6 ± 0.9 mg per egg mass obtained 3–6 days after oviposition), neolarvae (10 whole bodies pooled per replicate), nymphs (5 whole bodies pooled per replicate), males (unmated, 2 whole bodies pooled per replicate) and females (unmated, 1 whole body per replicate). All ticks (generation F81) were kept under the conditions described under section: “Tick rearing and animal care”.

Total RNA extraction, cDNA synthesis, qRT-PCR conditions, references genes and target gene (*Rhimi-PKR*) were the same used for and described in section RNA isolation, cDNA synthesis and quantitative reverse-transcriptase PCR analysis*.*

### *Rhimi-PKR* gene silencing by RNAi

#### In vitro RNAi evaluation of dsRNAs using the dual luciferase *Rhimi-PKR* reporter plasmid

It is critical to reduce the number of ineffective silencing dsRNAs tested on ticks placed on cattle due to the high cost, intensive labor and restrictions for the use of large animals in tick research. For these reasons, the silencing efficiencies of *Rhimi-PKR* dsRNA sequences were first tested in vitro in a dual luciferase system. The dual luciferase reporter was constructed for *R.* *microplus* as previously described [44] and subsequently adapted for in vitro assessment of RNAi efficacy targeting transcripts of *Rhimi-PKR*. The cDNAs cloned for dsRNA synthesis were amplified from messenger RNA isolated from whole unfed females of the Deutsch strain by RT-PCR using specific primers (Table 1). Primers were designed based on partial sequences derived from the *Rhimi*-*PKR*-cloned cDNA (KP126932 [23]), and from an additional 5’-untranslated region (UTR) sequence from the predicted transcript XM_037432703.1 (LOC119181450 pyrokinin-1 receptor-like *R.* *microplus*, southern cattle tick). RT-PCR was run with the Amplitaq Gold™ Kit (Applied Biosystems, Thermo Fisher Scientific) according to the manufacturer's specifications, using approximately 25 ng of template (cDNA) and at the following temperature-cycling parameters: 1 cycle of 95 °C for 5 min, followed by 35 cycles of 94 °C for 30 s, 55 °C for 30 s and 72 °C for 30 s, with a final cycle of 72 °C for 5 min. The PCR product fragments were then cloned into the *XmaI/SbfI* sites in two dual luciferase reporter plasmids using an InFusionTM cloning kit (Clontech™ Laboratories, Mountain View CA, USA). The first of these clones was RmPyr_DualLuc-5’: PK-584–580.2/pCR 2.1-TOPO, with a *Rhimi-PKR* cDNA insert of 895 bp that encompassed 197 bp of the 5’-UTR end of the KP126932 (in bold in Additional file 1: Figure S1), having an extended 5’-UTR sequence of 629 bp upstream of this section, and 69 pb of the open reading frame (ORF) of *Rhimi-PKR,* which was used to test the in vitro silencing activity of the ds762-913 (green highlight in Additional file 1: Figure S1). The second of these clones was DualLuc RmPyr#3’: PK-618.2/pCR 2.1-TOPO, with a *Rhimi-PKR* cDNA insert of 1638 bp that encompassed the majority of KP126932 as follows: 133 bp of the 5’-UTR end, the ORF, and 184 nucleotides (nt) at the 3’-UTR end, which was used to test the in vitro silencing activity of the ds1485-1627 (gray highlight in Additional file 1: Figure S1).

In vitro transfection of the *R.* *microplus* embryonic cell line BmE26 [45] was accomplished by using a mix containing Effectene transfection reagent (Qiagen, Hilden, Germany), 150 ng of the dualluc/pyrokinin construct (either RmPyr_DualLuc-5’ or RmPyr_DualLuc-3’) and 50 ng of the corresponding dsRNA to be tested for silencing activity: ds762-913 for construct RmPyr_DualLuc-5’ and ds1485-1627 for cells transfected with RmPyr_DualLuc-3’ [44].

Wells with no dsRNA and those with a dsRNA whose sequence does not overlap with the *Rhimi*-*PKR* sequence, designated dsfsg, which corresponds to *R.* *microplus* cholinesterase-like transcript (XM_037420972.1; see Additional file 1: Figure S1) were included in each assay as negative controls. At 5 days post-transfection, the cells and supernatant were harvested. The supernatant luciferase activity was measured using the Nano-Glo Luciferase System (Promega, Madison, WI, USA), and the cells were lysed and tested using the Steady-Glo Luciferase System (Promega). As the transfection with the cell line (BmE26) used in this assay is highly variable, the ratio of activity of the two luciferases was used as an efficiency parameter. The ratio of cell activity/supernatant activity was recorded for each well (Table 2). The dual luciferase reporter system uses two independently controlled luciferases, which allows one promoter to function as an internal reference standard. By calculating the ratio of expression of the two reporters, differences in relative transfection efficiency and dosage between wells are normalized with respect to the internal reference luciferase, allowing comparison of the normalized second luciferase activity [44]. Since the release of the *R.* *microplus* genome [6], further analysis of the specificity of *Rhimi-PKR* dsRNAs has become possible. To this end, for each *Rhimi-PKR* dsRNA sequence, the algorithm BLASTn was used in searches (https://www.ncbi.nlm.nih.gov/) to identify similar sequences in the genome (*R.* *microplus*, assembly ASM1333972v1) that could lead to off-target RNAi effects (Additional file 2: Figure S2).

**Table 2:** Luciferase activity and silencing efficiency obtained from *Rhipicephalus* *microplus* BmE26 cells at 5 days post-transfection with dsRNAs targeting the *Rhimi*-*PKR* sequence

Treatment set	Treatment applied to *R.* *microplus* PKR dual LUC-expressing cells	Size of dsRNA (bp)^a^	Luciferase ratio^b^	Silencing compared to no added dsRNA (%)^c^
Control (–)	No dsRNA	–	0.699827	0.0
Control (–)	dsRNA fsg	357	0.376921	22.9
1	dsRNA 762-913^d^	207	0.011161	98.3
2	dsRNA 1485–1627^d^	198	-0.039550	103.7

^a^Length of the dsRNAs in bp including the amplified sequences and the 5'- and 3’-ends T7 tags

^b^Luciferase ratio Steady-Glo/Nano-Glo

^c^Silencing = 1 − (luciferase ratio for dsRNA treatments/luciferase ratio control value [no dsRNA])

^d^dsRNAs used for the in vivo study

#### Synthesis of dsRNAs for RNAi

Target sequences for RNAi of 150–250 nt were selected from the *Rhimi-PKR* cDNA (KP126932.1) (Additional file 1: Figure S1), primarily from the 5’- and 3’-UTR regions. dsRNAs for the in vitro and in vivo gene silencing experiments were synthesized following the manufacturer’s instructions using the T7 RiboMAX™ Express RNAi System (Promega) and the clones and plasmids mentioned above. Oligonucleotide primer sequences (Table 1) were selected using the software Oligo v6.71 (Molecular Biology Insight Inc., Cascade, CO, USA) and adapted by the addition of the T7 polymerase recognition sequence, as specified by the manufacturer for dsRNA synthesis (Table 1). The concentration was determined using a Nanodrop 1000 spectrophotometer using optical density, A_260_/A_280_) ratios (Thermo Fisher Scientific). The dsRNA for the negative (beta-lactamase) and positive (*Rhimi-ACTB* gene) dsRNA controls were synthesized using the same conditions mentioned above, with the primers listed in Table 1. The *BLA* gene (beta-lactamase) encodes a bacterial enzyme that is not present in ticks. Beta-actin (used as a dsRNA positive control) is a gene that encodes for a ubiquitous protein in insects, involved in cell motility, structure, and integrity [46]. dsRNAs for both beta-lactamase (dsβ-lac) and beta-actin (dsβ-act) were previously used as a negative and positive controls, respectively, for silencing experiments in *R.* *microplus* [28, 47].

#### Microinjection of ticks

Ticks used in the RNAi experiments were unfed adult females, between 1 and 5 days after eclosion from collected nymphs, held in cotton-stoppered glass vials under constant temperature (25 ± 2 °C) and 95% RH conditions [43]. Four independent RNAi experiments were performed. Each replicate consisted of dsRNA-injected ticks and non-injected ticks (as negative control) held in round, cotton sleeves that were glued to the calves’ shaved backs [28, 48]. Each calf could accommodate up to eight sleeves, four on each side of the animal, and each animal was considered to be a randomized block. Each treatment corresponded to a sleeve, and treatments were randomly adjudicated to sleeves that were then labeled. The sleeves contained 35–40 females and 15–25 males. In total, five calves were used for these RNAi experiments.

Female ticks were microinjected with dsRNA specific for each treatment: dsRNAs for *Rhimi-PKR* (dsPKR) as the experimental group; dsβ-act as the positive controls; and dsβ-lac as the negative controls. These dsRNAs were diluted in 0.2 µl of NF-water at concentrations ranging from 5.21 to 7.5 µg/µl, with the exception of the dsβ-act, which was used at a dilution of 5.6–9.3 µg/µl (Additional file 7: Table S2), using methods previously described [49]. Injected ticks were moved to glass vials and kept at the same temperature and humidity conditions as with adult emergence (mentioned above) for 24 h post-injection, following which live and motile injected and non-injected females were transferred to the sleeves on the bovine host. The four independent biological replicates for each treatment were performed in 3 consecutive years: December 2019, March 2020 and July 2021. The delay between the second and third replicate was due to the SARS-COVID-19 pandemic causing the closure of the USDA-ARS facilities for animal experiments.

#### Tissue collection, RNA extraction, cDNA synthesis and qRT-PCR assays for evaluation of gene silencing

For evaluating silencing, we collected three partially fed females per treatment and biological replicate on the third and fifth day on the animal. The ticks that fed for 3 days were pierced and individually kept in 250 µl of RNAlater™ (Invitrogen) at − 80 °C until use. Ticks that were 5 days on the animal were dissected in cold physiological saline, and tissues were individually kept at − 80 °C until RNA isolation [28, 49]. RNAlater was used to store each carcass (250 µl) and ovary (100 µl). The synganglion was kept in 50 µl Trizol reagent (Invitrogen) to ensure RNA stability and because the synganglion is a very small tissue that becomes fully transparent in RNAlater and cannot be visualized for recovery. Ovary samples additionally included the vagina and seminal receptacle and likely muscles closely associated with these tissues. Samples were selected to analyze the silencing efficiency at the organism level (whole tick), and tissues were those in which a relative high expression for *Rhimi-PKR* transcript had been previously reported in *R.* *microplus* [23].

For the analyses of relative gene expression to evaluate the effect of the dsRNAs, total RNA extraction, cDNA synthesis, qRT-PCR variables, reference and target genes (*Rhimi-PKR* and *Rhimi-ACTB*) and preparation of reactions were the same as those described in section RNA isolation, cDNA synthesis and quantitative reverse-transcriptase PCR analysis*.*

To compare the relative gene expression among treatments, for the qRT-PCR assays all cDNA samples of non-injected, *Rhimi-PKR* (both dsRNAs 762–913 and 1485–1627) and dsβ-lac-injected ticks were loaded in the same 96 cell-well plate, and this was replicated in a total of three plates for each of the following: (i) ticks 3 days on the animal; (ii) carcasses; (iii) ovaries; and (iv) the synganglion of ticks that fed for 5 days. For each tissue (of replicates 1–4), relative expression was analyzed on two plates using primers for each reference gene (*Rhimi-EF1A* and *Rhimi-RSP4*), respectively, and on the third plate using primers for the gene of interest (*Rhimi-PKR*) and for *Rhimi-ACTB*. Therefore, a total of 48 plates were utilized (4 replicates x 4 tissues x 3 plates per tissue). The NRQ was calculated for *Rhimi-PKR* and *Rhimi-ACTB* following the protocol mentioned in section RNA isolation, cDNA synthesis and quantitative reverse-transcriptase PCR analysis.

The relative transcript abundance of *Rhimi-PKR* for each tissue was presented as a fold-change (FC) of dsβ-lac-injected ticks (FC = 1).

#### Phenotypic data collection

To register potential phenotypic changes associated with PKR silencing, each day after ticks were placed on the animals, the sleeves were opened, and ticks were photographed (Additional file 3: Fig. S3). Any detached engorged ticks found were removed, weighed, transferred to a cotton-stoppered glass vial and held under the laboratory maintenance conditions previously mentioned. The date of “drop” (self-detachment from the bovine) as well as the “repletion period” (duration of the female feeding period) were recorded. Engorged females in vials were monitored daily to record the date of the first oviposition to calculate the pre-oviposition period, which extends from detachment to oviposition. Because *R.* *microplus* females die after oviposition [42], the dead female tick was removed from the vial and the egg masses were weighed. Egg masses were monitored to determine the incubation period, which corresponds to the time lapsed from egg laying to first hatch. The percentage of emergence was used to calculate the egg hatch per female, which corresponds to the eggs that hatched per egg mass. This assay was performed by visual estimation by the same experienced technician for the duration of each experiment, as described in [50]. The overall time period from “attachment to animal” until hatching of the first egg for each female tick was also recorded as the cumulative “observation period.” The reproductive efficiency index (REI) = [(egg mass/replete female weight) × 100] was calculated for each female [51]. In addition, more phenotypes were recorded for each female, as defined: (i) female mortality was recorded over the course of the experiments; (ii) females with no eggs, calculated from the number of females that survived (these ticks were placed in vials, and did not lay eggs at all); (iii) females with no hatch, which reflects the percentage of produced egg masses that do not hatch at all.

Results for both PKR RNAi treatments ds762-913 and ds1485-1627 were compiled for the analysis of all phenotypic variables: mortality, repletion period, female weight, pre-oviposition period, egg mass weight, REI, females with no eggs, incubation period, egg hatching per female, females with no hatch and observation period. As per approved AUP that assures maintenance of the animal health and taking into consideration that the expected detachment time from the host for untreated ticks is 6–9 days, an endpoint for the experiment of 2 weeks after tick attachment to the calf was chosen. Ticks that did not detach before this pre-determined date were considered dead and not included in subsequent analyses. Further, the data obtained after the endpoint are highly variable, and tick death is often associated with factors such as fungus on the tick females, confounding results.

### Statistical analysis

Results of phenotypic variables and qRT-PCR assays were analyzed with and graphs were produced using GraphPad Prism v6 software (Graphpad Software Inc., San Diego, CA, USA). A one-way analysis of variance (ANOVA) followed by a Tukey’s HSD test was performed to compare PKR relative expression throughout developmental stages. The qRT-PCR relative expression data were similarly analyzed to verify PKR silencing in whole ticks and in different female tissues. Results are presented as the mean ± standard error of the mean (SEM). Kruskal–Wallis one-way ANOVA followed by a corrected Dunn’s multiple comparisons test was used to analyze phenotypic data, and the results are presented as the mean ± SEM.

## Results

### *Rhimi-PKR* expression throughout different stages of development

**Fig. 1:**
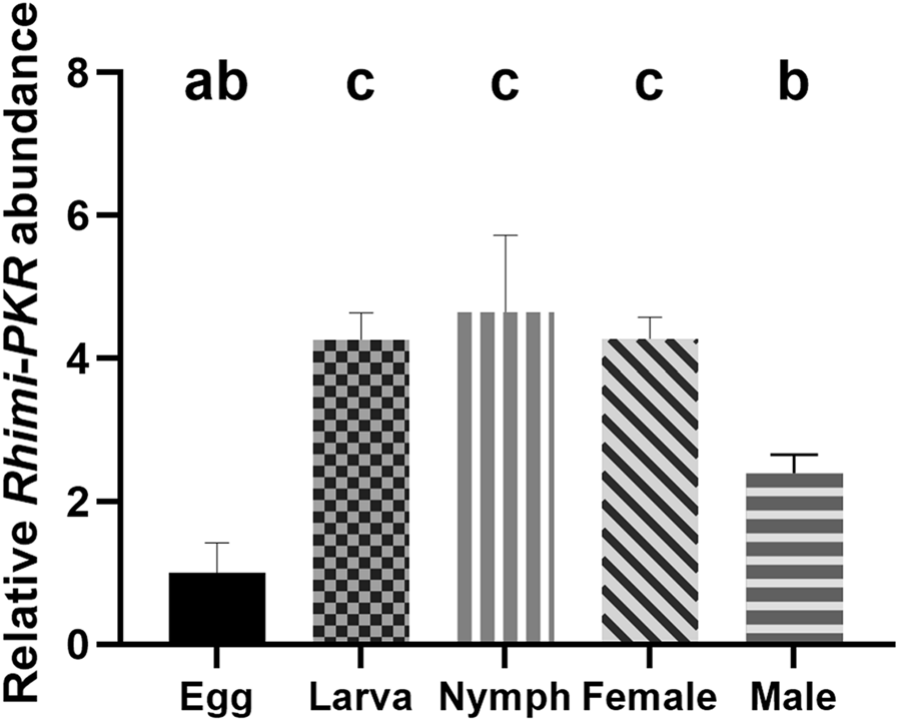
*Rhimi-PKR* relative transcript expression at different stages of development, measured by qRT-PCR. Stages of development analyzed were as follows using 8 biological replicates of each of the following stages: egg masses (Egg), neolarvae (Larva), nymph, male and female. qRT-PCR results showed that the *Rhimi-PKR* transcript was expressed throughout all stages of development, with the lowest expression observed for eggs and males. The relative *Rhimi-PKR* abundance for eggs was used as the reference ratio (calibrator) for the FC calculations. *Rhimi-PKR* abundance was similar for neolarva (Larva in histogram), nymph and female, and higher than in egg by FC factors of 4.3, 4.7 and 4.3 (*n* = 8 biological replicates; *P* < 0.05), respectively. *Rhimi-PKR* abundance for male was the second lowest, and not different from that in egg (*n* = 8; *P* > 0.05). A one-way ANOVA followed by a Tukey’s multiple comparisons test was used for the statistical analysis. Different lowercase letters at top of histogram bars indicate significant difference. Abbreviations: ANOVA, Analysis of variance; FC, fold change; qRT-PCR, quantitative reverse-transcriptase PCR; *Rhimi*-*PKR*, *Rhipicephalus microplus-pyrokinin receptor*

The qRT-PCR analyses showed that the *Rhimi-PKR* transcript was expressed throughout all stages of development of *R.* *microplus* (Fig. 1). The lowest *Rhimi-PKR* relative expression was observed for egg and male (Fig. 1), and the relative abundance for egg was used as the reference ratio (calibrator) for the FC calculation. *Rhimi-PKR* relative abundance was similar for larva, nymph and female, surpassing the expression in eggs by a factor of 4.3–4.7 (*n* = 8 replicates; *P* < 0.05) (Fig. 1). *Rhimi-PKR* relative abundance for male was not significantly different from that of egg (*n* = 8; *P* > 0.05) (Fig. 1).

### RNAi silencing of *Rhimi-PKR*

**Fig. 2:**
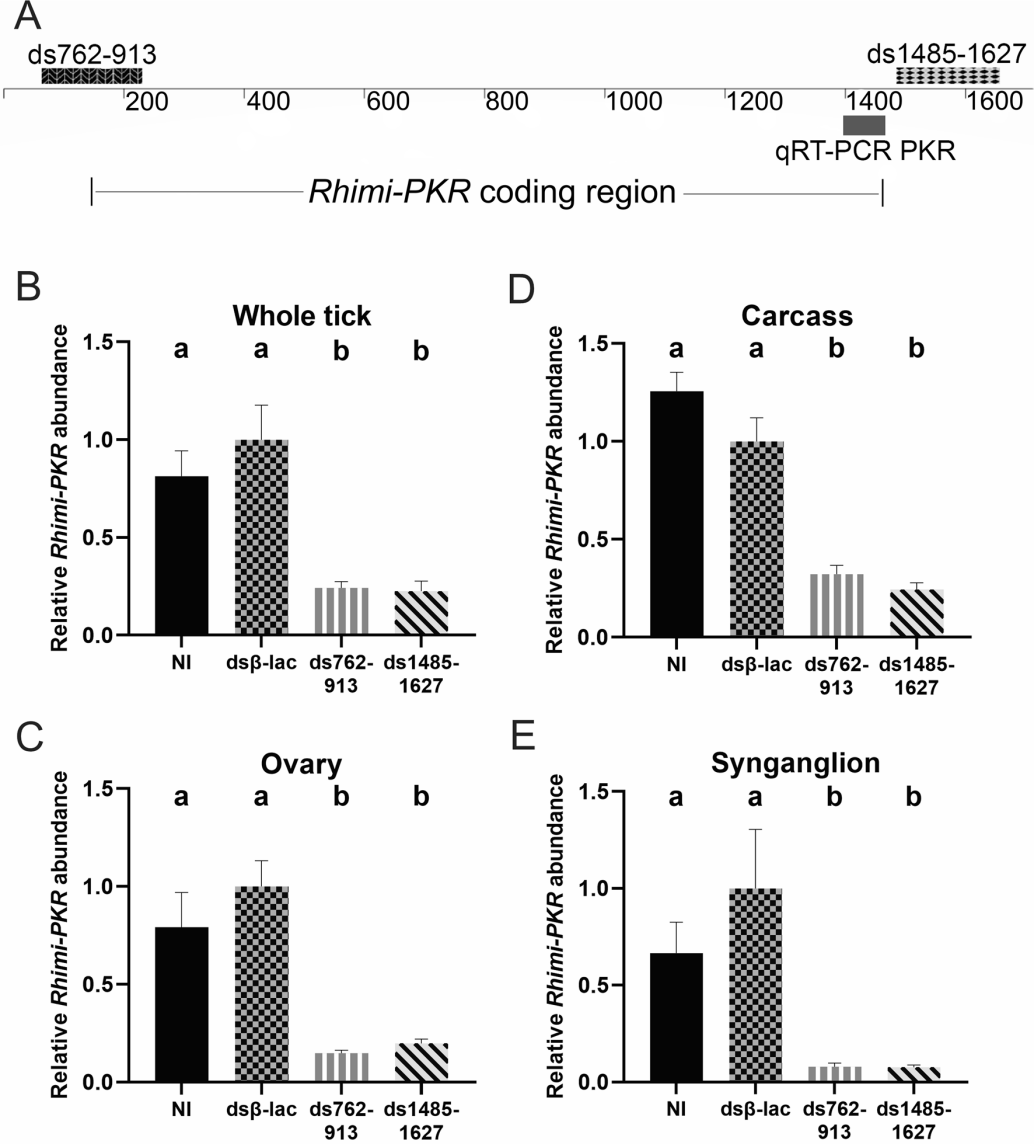
RNAi silencing of *Rhimi-*PKR*.* **a** Location of the PKR dsRNAs and qRT-PCR amplicon on *Rhimi-PKR* full-length cDNA sequence [23]. The full-length cDNA sequence is 1713 bp with a 1326-bp ORF (KP126932.1), which is depicted limited by vertical bars. Locations of PKR dsRNAs are highlighted as a darker box on the left for ds762-913, and as a light gray, black-dotted box for ds1485-1627, on the right. The PKR qRT-PCR amplicon is shown as a solid-gray box, below. An additional 5’-UTR sequence beyond the original cDNA cloned sequence was obtained based on the genomic sequence (not shown). The 5’-dsRNA762-913 identification numbers refer to the nucleotide positions that encompassed on that additional 5’-UTR sequence (Additional file 1: Figure S1, alignment of all PKR sequences), thus the numeric discrepancy between the cDNA clone positions and the ds762-913 shown on the left of the schematic. The ds762-913 nucleotide position in the cDNA KP126932.1 is from − 65 to + 66 nt. For more details see section In vitro RNAi evaluation of dsRNAs using the dual luciferase Rhimi-PKR 236 reporter plasmid and Table 1. **b-e** qRT-PCR analyses of partially fed female ticks from all treatments to confirm *Rhimi-PKR* silencing: **b** whole tick, 3 days on calves; **c **ovary, **d** carcass **e** synganglion, from females 5 days on calves. Primers used for the analysis are given in Table 1. A one-way ANOVA followed by a Tukey’s multiple comparisons test was used for the statistically analysis. Different lowercase letters at top of histogram bars indicate significant difference (*P* < 0.05). Abbreviations: cDNA, Complementary DNA; ds, double-stranded; ORF, open reading frame; UTR, untranslated region

For the RNAi experiment, two dsRNAs, designated ds762-913 and ds1485-1627, respectively, were tested in vivo (Fig. 2a). The dsRNAs were considered suitable for in vivo experiments on the basis that they efficiently silenced the *Rhimi-PKR* transcript in the dual luciferase reporter assay (see section In vitro RNAi evaluation of dsRNAs using the dual luciferase Rhimi-PKR 236 reporter plasmid) (Table 2). To test for possible off-target effects, we conducted BLASTn searches, but only sequences ≤ 17 nt were found with similarity to *Rhimi-PKR* dsRNA sequences (Additional file 2: Figure S2).

For in vivo experiments, silencing efficiencies of dsRNAs are reported with respect to the dsβ-lac-injected females. qRT-PCR analyses showed that injections with ds762-913 caused a mRNA *Rhimi-PKR* knockdown of 76% (average for the 4 replicates) in whole ticks that were 3 days on the animal (Fig. 2b). Females injected with the same dsRNA that were dissected after 5 days on the animal showed a decrease in *Rhimi-PKR* transcript in the ovary, carcass, and synganglion of 85, 68, and 92%, respectively (*n* = 12 (4 replicates x 3 ticks or tissues), *P* < 0.05; Figs. 2c–e). For females injected with ds1485-1627, the mRNA of *Rhimi-PKR* showed a decrease of 77% (average for the 4 replicates) in whole ticks that were 3 days on the animal (Fig. 2b). For those ticks dissected after 5 days on the animal there were reductions in the ovary, carcass, and synganglion of 80, 76, and 92%, respectively (average for the 4 replicates) (Figs. 2c–e). The *Rhimi-PKR* silencing results for ds762-913 and ds1485-1627 for each independent replicate are shown in (Additional file 4: Figure S4), and both dsRNAs were equally effective in silencing the gene in all four replicates, and for both the whole tick and tissues. These sequences were also verified in the available *R.* *microplus* genome at NCBI [*Rhipicephalus* *microplus* (assembly ASM1333972v1)/locus LOC119181450] (Additional file 2: Figure S2). The average silencing efficiency for the positive control, dsβ-act, for the four replicates in the whole tick, carcass, ovary and synganglion was 91, 97, 94 and 96%, respectively (Additional file 5: Fig. S5).

### Phenotypes associated to the RNAi silencing of *Rhimi-PKR*

**Fig. 3:**
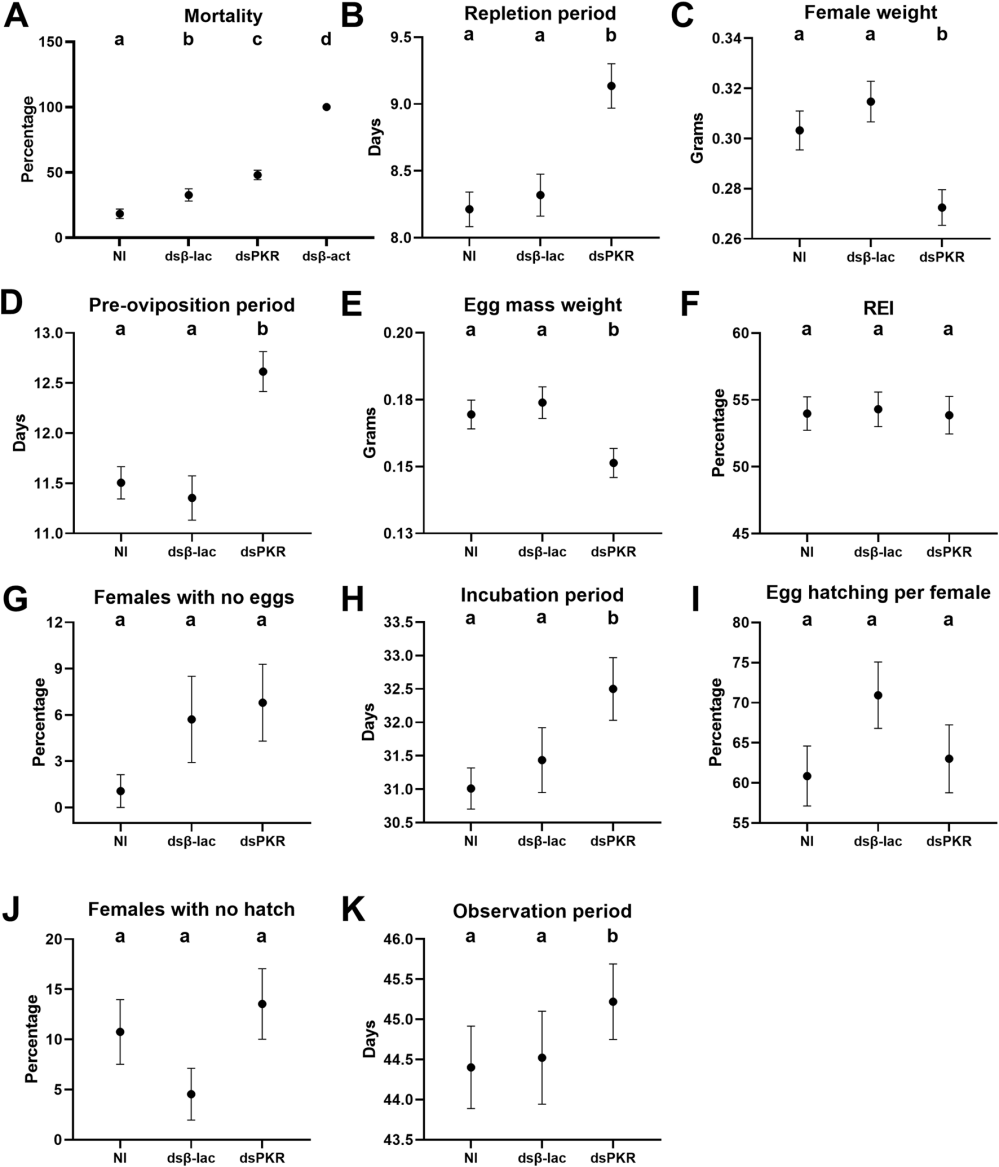
Phenotypic evaluation of* Rhimi-PKR* silencing in* Rhipicephalus microplus* females. Results for both treatments, PKR ds762-913 and ds1485-1627 were compiled for the analysis of all the phenotypic variables: **a** percentage of female mortality, **b** duration of repletion period, **c** female weight, **d** duration of pre-oviposition period, **e** egg mass weight, **f** REI, which represents the conversion of the blood intake to egg mass production, **g** females with no eggs, i.e. females that showed not oviposition at all, **h** duration of egg incubation period, **i** percentage of egg hatching per female, **j** females with no hatch, i.e. percentage of females whose egg mass had no eggs hatching at all, **k** total observation period, which represents the time period from “tick attachment to animal” until hatching of the first egg for each female. Positive control: *Rhimi*-*ACTB* dsRNA-injected ticks (dsβ*-*act). Treatments sharing the same lowercase letter (top of each graph) were not significantly different from each other (*P* < 0.05) as determined by the Kruskal–Wallis test followed by the corrected Dunn’s multiple comparisons test. Abbreviations: NI, Negative controls (non-injected ticks); dsβ-lac, beta-lactamase dsRNA-injected ticks; dsPKR, *Rhimi-PKR* dsRNA-injected ticks; REI, reproductive efficiency index

**Fig. 4:**
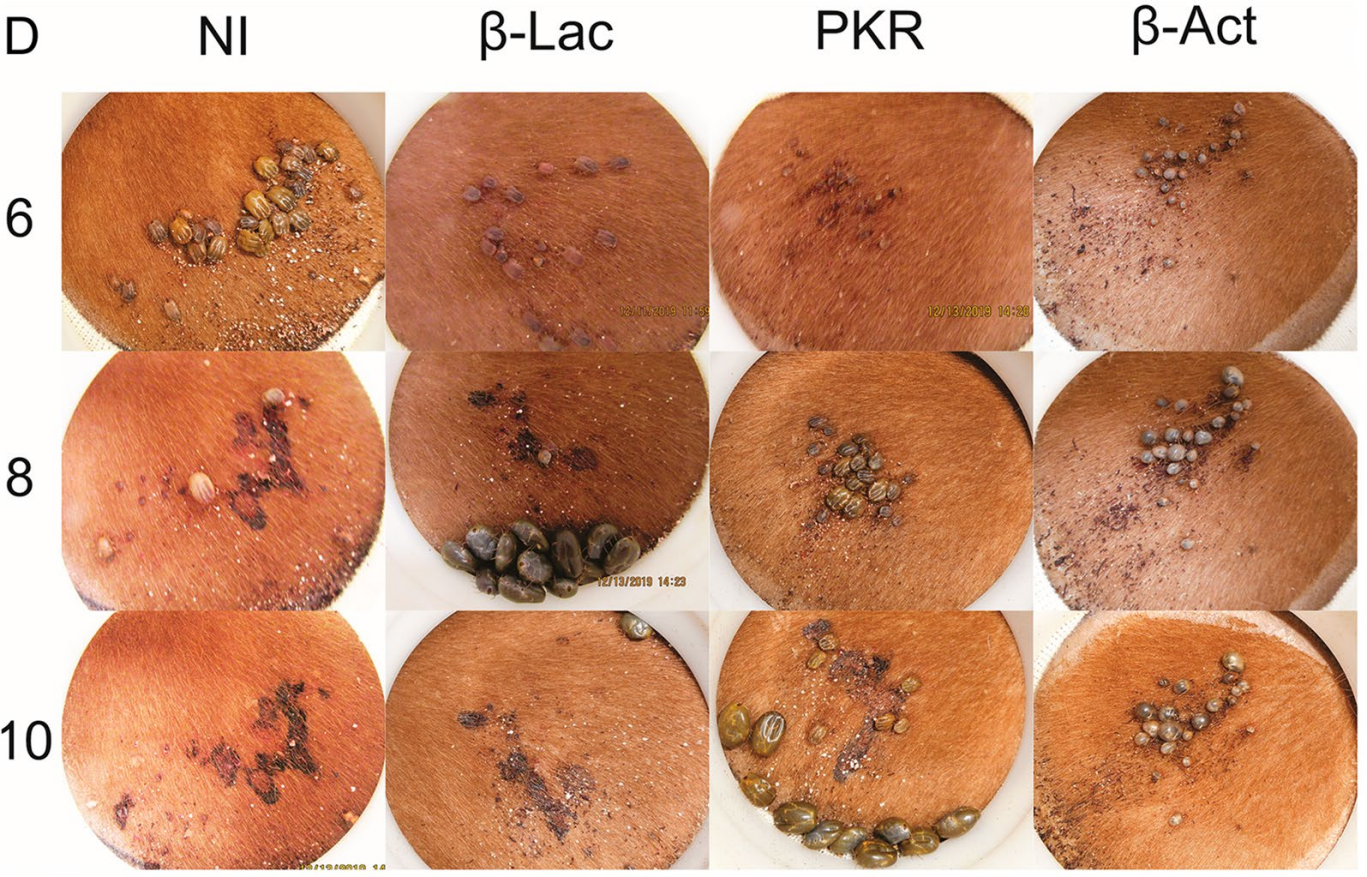
Feeding progression of *Rhimi-PKR* silenced ticks. Photographs of open cotton sleeves showing shaved patches on cattle where the confined *R.* *microplus* female ticks fed, photographed at 6, 8 and 10 days (numbers on the left side of the figure). Additional pictures of ticks feeding throughout the course of the experiment are shown in Additional file 3: Figure S3. Abbreviations: D, Days feeding on the animals; NI, non-injected ticks; β-Lac, beta-lactamase dsRNA-injected ticks (negative controls); PKR, *Rhimi-PKR* dsRNA-injected ticks; β*-*Act, *Rhimi*-*ACTB* dsRNA-injected ticks (positive control)

More than 95% of the ticks that were placed on the calves from all treatments dropped off and were collected within the 2 weeks of the experiment. After specific *Rhimi-PKR* silencing was verified by qRT-PCR (Fig. 2), the phenotypic traits of ticks under each treatment were evaluated; the results at the endpoint of the assay are summarized in Fig. 3. Tick pictures representative of the treatment effects after 6, 8, and 10 days on the calves are shown in Fig. 4. A complementary detailed record of the progression of tick sizes for all treatments in days 6 to 10 is shown in Additional file 3: Figure S3.

dsPKR-injected ticks showed an increased net mortality of 15% (*n* = 110–198; *P* < 0.05) relative to the dsβ-lac-injected females (negative controls) (Fig. 3a). All dsβ-act-injected females (positive control) died, as expected, and were not included in the remaining analyses (Fig. 3a). The lowest mortality was for non-injected ticks (18.3%), followed by dsβ-lac-injected ticks with 32.7% mortality (Fig. 3a).

The repletion and pre-oviposition periods were 0.8 (*n* = 103–125; *P* < 0.05) and 1.3 (*n* = 96–118; *P* < 0.05) days longer, respectively, for dsPKR-injected female ticks in comparison to dsβ-lac-injected females (Fig. 3b, d). There were no significant differences between the negative control treatments for both traits. In addition, dsPKR-injected females had a net decreased weight (*n* = 103–123; *P* < 0.05) and net decreased weight of the egg masses (*n* = 96–118; *P* < 0.05) of 13% for both phenotypic traits relative to dsβ-lac-injected females (Fig. 3c, e, respectively). Similarly, there were no differences between the negative control treatments for both traits. However, the REI, which reflects the percentage of conversion of female weight to egg mass (*n* = 96–119; *P* > 0.05), showed no difference between dsPKR-injected ticks and negative controls (Fig. 3f).

With respect to the number of females with no eggs (live females that did not oviposit), no significant statistical differences were observed (*n* = 103–123; *P* > 0.05) among the three treatments (Fig. 3g). On the other hand, the incubation and observation periods were significantly longer by 1.1 (*n* = 82–107; *P* < 0.05) and 0.7 (*n* = 82–107; *P* < 0.05) days, respectively, for dsPKR-injected female ticks (Fig. 3h, k). The non-injected ticks did not differ from the dsβ-lac controls for both traits (Fig. 3h, k).

Finally, for the percentage of egg hatching per female (*n* = 84–107; *P* > 0.05) and the number of egg masses that showed no hatch at all (females with no hatch) (*n* = 82–107; *P* > 0.05), there was no statistical difference between dsPKR-injected ticks and negative controls (Fig. 3i, j).

Representative phenotypic image records of ticks for all treatments in patches for days 6, 8 and 10 after being placed on the animals are shown in Fig. 4. These phenotypic images accurately reflect the results of the phenotypic traits quantitatively evaluated in Fig. 3, but further provide visual evidence of the differential feeding progression for all treatments. Many of the non-injected ticks (NI) fed to repletion between days 6 and 8, and by day 10 most of them had dropped from the animal. For the dsβ-lac-negative controls, while the records appear to indicate a delay in their repletion with respect to the NI (compare day 8 NI, with β-Lac; Fig. 4), there was no significant difference between the two treatments in terms of repletion period by the endpoint (Fig. 3b); it should be noted that most of them had similarly dropped off the animal by day 10. In contrast, the PKR-silenced ticks that survived appear to be repleted by day 10, significantly delayed in their repletion period (Fig. 4 PKR; Fig. 3b) and reached a significantly lower final weight (Fig. 4 PKR; Fig. 3c). The dsβ-act-injected ticks showed a rounded shaped body at day 6 (compare NI with β-Act), and although some appeared to continue to feed by day 10 while others appear to have reduced their size by the same day, all of them died by the endpoint of the assay (Fig. 3a), and none fed to repletion (compare day 6 NI, day 8 β-Lac and day 10 PKR, with β-Act).

## Discussion

Pyrokinins are pleiotropic neuropeptides in insects, and one of their functions is to promote muscle contractions [8]. While we recently reported that pyrokinins and a pyrokinin peptide analog elicit the contraction of feeding-related tissues of *R.* *sanguineus* in vitro [25], the pleiotropic functions of the pyrokinin signaling system in ticks are still unexplored. Therefore, we performed loss-of-function experiments by RNAi of the pyrokinin receptor in live females of *R.* *microplus* to further investigate the physiological significance of the pyrokinin signaling system in ticks and to assess if this GPCR could be a good candidate for control interventions (e.g. acaricides or vaccines).

RNAi has proven effective in inducing specific gene silencing in *R.* *microplus* [28, 48, 52–54]. Previously, we established that the receptor transcript was expressed in female tissues [23]. In the present work, we determined that the PKR transcript was also expressed in male ticks and was present throughout all stages of development of *R.* *microplus* (Fig. 1), which would be crucial for a candidate control target. Several research studies have looked at the possibility of designing dsRNAs in the 5’-UTR or 3’-UTR [55]. The advantage of choosing these regions is that they are less conserved than the coding sequence, leading to much higher specificity [55]. Selected regions in the 5’- and 3’-UTRs of the PKR cDNA sequence were chosen as targets for RNAi, and both were found to be equally effective. These results are similar to those on the pea aphid, *Acyrtosiphon* *pisum*, in which no differences in efficiency were found between dsRNA targeting the 5’- and 3’-UTR ends of the *hunchback* gene [56]. However, to discard possible off-target effects, we first ran BLASTn searches against the *R.* *microplus* genome. The results of these searches indicated that *Rhimi-PKR* dsRNAs off-target effects are highly unlikely, since 17 bp in length was the maximum contiguous identical sequence for the alignment between *Rhimi-PKR* dsRNA sequences and non-target sequences (Additional file 2: Figure S2). These alignments are too short to trigger meaningful off-target effects according to the authors of previous studies who concluded that a sequence of 19–21 nt of contiguous identical sequence is required to produce significant biological activity in RNAi-sensitive insect species [55, 57].

In the present study, we used a successful RNAi protocol to characterize the PKR, a GPCR of *R.* *microplus*. This protocol was previously successful for RNAi of the kinin receptor, another neuropeptide tick GPCR [28]. The protocol included first the use of a dual-luciferase reporter system to validate the silencing efficiency of dsRNA in vitro, prior to performing in vivo experiments with ticks on cattle that was developed by [44]. These in vitro assays were performed to increase the probability of success and specificity of the RNAi, being cognizant that the molecular components of the RNAi pathway(s) are still being characterized in the Acari [58]. RNAi experiments in *R.* *microplus* are particularly expensive and difficult to conduct beyond the normal complexity of performing RNAi in ticks [59, 60] because this is a one-host tick and a quarantined species in the USA, and because APHIS-approved cattle facilities are required [28]. Both dsRNAs were highly effective in silencing the PKR transcript in the dual-luciferase assay (Table 2). We then performed PKR RNAi in female ticks using these validated dsRNAs. qRT-PCR analyses demonstrated that both dsRNAs were effective in silencing the receptor in vivo (Fig. 2b-e), and silencing was detected in whole ticks 4 days after injection (Fig. 2b), and in the ovary, carcass and synganglion of females after interference that had been 5 days on the calves (Fig. 2c-e). The significant silencing of the PKR transcript in vivo verified by qRT-PCR underscores the importance of verification by the in vitro system before studies are undertaken in ticks using cattle.

The results point to the direct or indirect involvement of the PKR signaling system in the regulation of female feeding and reproductive output in *R.* *microplus*. Through the *Rhimi-PKR* silencing by RNAi, two physiological processes related to feeding were affected: the repletion period was delayed, and female weight decreased (Fig. 3b, c). With respect to reproductive output, the pre-oviposition and incubation periods were longer, suggesting an overall delayed egg/embryo maturation (Fig. 3d, h). Moreover, the egg mass weight was lower in comparison to the controls (Fig. 3e). A *R.* *microplus* female produces a compact egg mass of about 3000 eggs [42]. Since the percentage of eggs hatching was not affected by *Rhimi-PKR* silencing (Fig. 3i), we hypothesize that the decrease in the weight of egg masses was due to the reduction in the number of eggs (quantity) rather than the quality of the eggs; however, eggs were not counted. Overall, *Rhimi-PKR* silencing increased female mortality and reduced the fitness of surviving females, which is reflected in the delayed observation period that encompasses the female feeding and the day of first egg hatching (Fig. 3a, k).

Based on the results of this study, our recent work [25] and previous evidence in insect tissues reviewed in [9], the PK tick signaling system appears to activate muscles involved in feeding. Unlike blood-feeding insects that feed through blood vessels, ticks are pool feeders, with a complex and active feeding mechanism [61]. The feeding process involves host skin penetration by the hypostome, retraction of the cheliceral shafts and lateral movement of cheliceral digits [61]. The blood is sucked into the food canal and pharynx by contraction of the dilator muscles of the pharynx. Relaxation of these muscles allows the blood to pass through the esophagus into the midgut [62]. Consequently, a reduced activity of one or more of these muscles will result in a decreased feeding performance, in agreement with our results. *Rhipicephalus* *microplus* ticks cement themselves to the host, and once attached, the female takes a prolonged meal that is characterized by two periods: the “slow phase,” which occurs during the first 7–8 days, with a fed:unfed weight ratio of 10:1, and the “rapid phase,” which represents the 12–24 h during which there is an additional tenfold weight increase [63]. Our results support the hypothesis that the feeding process (one or both, slow and rapid phases) was affected by the interference of the pyrokinin signaling system: a delayed feeding progression (Fig. 4), a longer repletion period (Fig. 3b) and a decreased female weight (Fig. 3c). The photographs documenting feeding progression suggest that the slow phase is affected (compare day 8 β-Lac vs. PKR) (Fig. 4), but further studies are needed to determine if this or both phases were compromised. The hypothesis is further supported by our recent study that demonstrated the myotropic activity of PK in feeding-related tissues of two tick species, *R.* *sanguineus* and *I.* *scapularis* [25], being the first report to explore the functional activity of PK and a PK analog in ticks. Additional evidence supports that the pyrokinin signaling system is associated with the feeding-related tissues in arthropods. *Drosophila* *melanogaster* pyrokinins are encoded by the capability (*CAPA*) and *hugin* genes, respectively [64, 65]. In *Drosophila* larvae *hugin*-expressing neurons send projections to the pharyngeal muscles [66, 67], and Schlegel et al. [68] observed that hugin neuropeptides are associated with feeding regulation. In Lepidoptera, PK/PBAN is expressed in the subesophageal ganglion (SOG) [69], and several studies support the SOG as a taste and food intake control center in insects (reviewed in [70]). Consequently, we propose that the decreased reproductive output and the overall female fitness reduction observed after *Rhimi*-*PKR* silencing are consistent with defects in feeding.

There is some evidence that PKR transcript expression in tick reproductive tissues may be positively correlated with feeding. In unfed females of both *I.* *scapularis* [24] and *R.* *sanguineous* [25], the relative expression of the PKR is significantly higher in the synganglion, with reproductive tissues having lower relative expression. In contrast, in *R.* *microplus* partially fed females, the highest relative transcript expression is in reproductive tissues, followed by the synganglion [23]. These findings not only support a possible direct functional role for PK on the female reproductive tissue in ticks, but also, possibly, in a feeding-dependent manner. The quantity and quality of food intake are related to ovary development and egg maturation in insects [71, 72]. In the present work, the pre-oviposition and incubation periods were delayed, and the egg mass weight decreased after *Rhimi-PKR* silencing. These results could be associated to a decreased blood intake (Fig. 3b, c), or a possible reduction of PK activity in the reproductive tissues themselves, due to a decreased PKR expression and signaling in the latter.

In lepidopterans, diapause hormone (DH; a PK/PBAN family peptide) initiates embryonic diapause in the silkworm *Bombyx* *mori* Linnaeus [73, 74] however breaks pupal diapause in *Heliothis* and *Helicoverpa* spp. [18, 75–77]. Further, pupal diapause duration is modulated using DH analogs, both agonists and antagonists in *Helicoverpa* *zea* [78, 79]. The sequence WFGPRLa is the conserved C-terminal motif for the DH in insects [8]. None of the *R.* *microplus* predicted pyrokinins contain this exact sequence [25]. However, Suwan et al. [74] observed diapause induction in eggs after injecting *B. mori* pharate adults with DH-like peptides of varied length featuring the C-terminal motif **F**X**PRLa** (i.e. W**F**G**RPLa** and C**F**G**PRLa**), characteristic of the PK/PBAN/DH neuropeptide family [8]. The Rhimi-CAPA-PK2 is the only predicted PK from the cloned cDNA of *R. microplus* featuring the conserved motif in its sequence GT**F**V**PRLa**, ending in RLa while the other PKs end in RNa or RIa [25]. The Rhimi-CAPA-PK2 activates the recombinant *R.* microplus PK receptor with an EC_50_ of 188 nM [30], therefore, it is expected to similarly activate the receptor in vivo. The identical sequence is present in *R.* *sanguineus* (Rhisa-CAPA-PK2), while this peptide in *I.* *scapularis* is GS**F**V**PRLa**, Ixosc-CAPA-PK2 [25]. After PK receptor loss of function in *R. microplus* females, we observed a delay in egg incubation period. 

Several types of diapause have been observed in Prostriata and Metastriata ticks [80–83], including a delay of oogenesis in engorged females and a delay in the onset of embryogenesis in eggs [81]. Several factors are associated with the induction of developmental and behavioral diapause in ticks, such as daylight, temperature (high or low), starvation and humidity [80, 81]. In *Ixodes* *ricinus* Linnaeus (Acari: Ixodidae), Belozerov observed that diapause induction in the unfed female was manifested as delayed embryogenesis within the eggs, rather than as delayed oogenesis [81]. In addition, a study conducted in *D.* *silvarum,* showed that egg masses laid by post-diapause females were significantly smaller than those laid by females that did not experience diapause [84], suggesting a correlation between female diapause and the reproductive output. PKR presents a similar expression pattern and a conserved myotropic functional activity in insects and ticks. In ticks, the PKR transcript is expressed in the female reproductive tissue [23, 24], and we observed a decrease in the weight of the female and egg masses, as well as delayed pre-oviposition and incubation periods after *Rhimi-PKR* silencing. Regarding hormonal control of diapause in ticks, the breaking of diapause in larvae of *R.* *sanguineus* and *Dermacentor* *albipictus* Packard (Acari: Ixodidae) was observed after ecdysteroid treatment [85, 86]. In addition, Khalil also suggested that the induction of female diapause in *Argas* *arboreus* Kaiser (Acari: Argasidae) is associated with a decrease in a gonadotropin hormome, which is regulated by an unknown hormone secreted by the synganglion [87]. Based in our results, a role for PK in the regulation of oviposition and/or embryonic development warrants exploration in *R.* *microplus*. CAPA-PK2 peptides ending in **F**X**PRLa **should be investigated for induction or breaking of tick diapause.

*Rhimi-PKR* silencing increased female mortality by 15% versus dsβ-lac-injected ticks (Fig. 3a). Previously, increased mortality in *D.* *melanogaster* [88] and the moths *H.* *zea* and *Heliotis* *virescens* [89, 90] was reported following the application of PK/PBAN analogs and antagonists. The results on *D.* *melanogaster* [88] were obtained under stress conditions, and the authors suggested that the mortality could be associated with a deregulation of the water/ionic balance. PKR transcript expression has been detected in both the midgut-hindgut and Malpighian tubules in *R.* *microplus* and *I.* *scapularis* [23, 24]. Thus, PK could additionally have a role in fluid homeostasis regulation in ticks.

## Conclusions

The RNAi results of the present study suggest that the PK signaling system is an attractive target for the use of PK antagonists for tick control because the loss of receptor transcript expression was detrimental to females. We had previously characterized the PKR from the cattle fever tick *R.* *microplus* [23]. Recently, we demonstrated the myotropic activity of PKs in feeding-related tissues of two tick species, *R.* *sanguineus* and *I.* *scapularis* [25], and with the present work, we took a further step in the characterization of the PKR transcript in ticks. These results support a role for PK neuropeptides throughout all stages of tick development and in the regulation of tick female feeding and reproduction. It appears that PKs may have pleiotropic functions in ticks, similar to what they have in insects.

## Supplementary Information

**Additional file 1: Figure S1.** Nucleotide sequences and alignment of *Rhimi-PKR* (KP126932.1) and the clones RmPyr_DualLuc-5’ and RmPyr_DualLuc-3'. These two clones were used for the *Rhimi-PKR* in vitro silencing using *R.* *microplus* BmE26 cells.

**Additional file 2: Figure S2.** NCBI-BLASTn searches to check for possible off-target effects of the PKR dsRNA sequences.

**Additional file 3: Figure S3.** Pictures of ticks showing the feeding progression from day 3 to 15 for all treatments.

**Additional file 4: Figure S4.** Results of qRT-PCR evaluating RNAi silencing of *Rhimi-PKR* shown for the independent replicates. A one-way ANOVA followed by a Tukey’s multiple comparisons test was used for the statistical analysis. Different lowercase letters indicate significant difference (*P* < 0.05). Abbreviations: NI, Non-injected; β-lac, beta-lactamase dsRNA-injected ticks (negative controls); ds762-913 and ds1485-1627, *Rhimi-PKR* dsRNA-injected ticks; W tick, whole tick; Syn, synganglion.

**Additional file 5: Figure S5.** Verification of *Rhimi-ACTB* silencing as suitable positive control by qRT-PCR, for all replicates combined. A one-way ANOVA followed by a Tukey’s multiple comparisons test was used for the statistical analysis. Different lowercase letters in the figure indicate significant difference (*P* < 0.05). Abbreviations: NI, Non-injected; β-lac, beta-lactamase dsRNA-injected ticks (negative controls); dsβ*-*act, *Rhimi-ACTB*, dsRNA-injected ticks (positive control).

**Additional file 6: Table S1.** Analyses of the stability of reference genes using BestKeeper, Normfinder, Genorm and the comparative Delta-Ct method software tools, performed on randomly selected samples from the present work. Sheet 1 shows the comprehensive analysis of gene stability (which simultaneously assesses the results of each method) that determined that *Rhimi*-*RPS4* and *Rhimi-EF1A* were the most stable genes. This sheet also includes the average of three technical (pseudo)replicates for each gene and sample evaluated. Sheets 2 to 5 include the independent analysis of each method (BestKeeper, Normfinder, Genorm and Delta-Ct).

**Additional file 7: Table S2.** dsRNA treatments and respective concentrations used in each replicate of the RNAi experiment.

## Data Availability

All relevant data are provided in the manuscript.

## Abbreviations

AUP: Animal use protocols
cDNA: Complementary DNA
CFTRL: Cattle fever tick research laboratory
DNAse 1: Deoxyribonuclease 1
FC: Fold-change
GPCR: G-protein-coupled receptor
NF-water: Nuclease-free water
nt: Nucleotides
OD: Optical density
PK: Pyrokinin
PK/PBAN/DH: Pyrokinin/pheromone biosynthesis-activating neuropeptide/diapause hormone
PKR: Pyrokinin receptor
qRT-PCR: Quantitative reverse-transcriptase PCR
REI: Reproductive efficiency index
RNAi: RNA interference
RT: Room temperature
SD: Standard deviation
SEM: Standard error of the mean
SOG: Subesophageal ganglion
USDA-ARS: United States Department of Agriculture-Agricultural Research Service
UTR: Untranslated region
