# Supplementary material for: Pyrokinin receptor silencing in females of the southern cattle tick Rhipicephalus (Boophilus) microplus is associated with a reproductive fitness cost
Source: Parasit Vectors. 2022 Jul 11;15:252. doi: 10.1186/s13071-022-05349-w (PMC9272880; doi:10.1186/s13071-022-05349-w)
Supplement: Supplementary file 1 — Additional file 1: Figure S1. Nucleotide sequences and alignment of Rhimi-PKR (KP126932.1) and the clones RmPyr_DualLuc-5’ and RmPyr_DualLuc-3'. These two clones were used for the Rhimi-PKR in vitro silencing using R. microplus BmE26 cells. [file 13071_2022_5349_MOESM1_ESM.docx]

Start and stop codons are highlighted in red.

***Rhimi-PKR* (GenBank KP126932.1) 1713 bp**

TTCGAGCCGGAAAGAGTTCTTAGTGCCCCTTCATCTCCTCTTTTCTGCTATATCAAAACGCAGCGAGAGG

AGCCTCAACGAGACGGCCACAATGGAACTGTCAAGCAGGCTGAGGCAGATCGGATCCCAATCTGAGACCG

GAGGAGC**ATG**ATCCCACCACAGCCGGCGTCCACCCTGGCGCACACGGACGCTGCGACCGACATGGCTTCC

GCCGACGAAGACGACACCGGTACGCAGGCCGACGATCCCCTGGTGACGCTGAACGCGTCAGCAGCCGCCG

AAATGCTCCTCCTGGCATTGGGCCCCAAGCGCGACCCGCTAACCACGGTCATCCCAATGACGCTCATCTA

CTCTGTCCTGCTCGTCTCCGGAGTGGTCGGCAACGTCTGCACGTGCATCGTCATCGCGCACAACCGGTAC

ATGATGCACACGGCGACCAACTACTACCTCTTCAGCTTGGCCGTCTCCGATCTGCTCCTGCTGGTGCTCG

GGCTGCCCCAAGAACTGTACCAACTCTGGCAGCGGCACCCGTACGTGTTCGGAGAGGCATTCTGCGTGCT

CCGGGGCCTGACCTCGGAGACCTCAACCAACGCGTCCATACTGACCATCACCGCGTTCACGATCGAGCGC

TACGTGGCGATCTGCCACCCGCTGCGGGCCCACACCATGTCCAAGTTGTCCCGGGCCGTCAAATTTGTCG

TCGCCATTTGGGTCCTAAGCGCCGTGTGTGCCGTCCCGCTCGCCGTCCAGTTCGGCATCGTCCACCAGAC

GCTGGACGGCACGACGGTGCTTCCGGAGACGGCCGCGTGCACGGTCAAGGACCCGCTGGAACACGCCTTC

GAACTGTCCACGTTCGTGTTCTTCTTGCTGCCCATGAGCGTCATTCTGGTGCTGTACGTTTGCATAGCGC

TGCAGCTGAAGCGCTCCGACGCCCTGTCGCGCCGGGACGTGCACCACAAGTGTCCCGCCTCGAACAGCCC

CAGCACCAGTGTCGTCAACGGGAAGGGCGACTCCTCGTCCACCGTGGGGCACAAACAGAGTTCGGCTGTG

GTGCAGCCGCTGCCCAGCAAGCTGCAGAGGGGCTGCCAGCTGAGGAAATCGGTTCGCGGAGGTGCGGCCG

CGTCATCGTCCCGCAAGGCGGTCATCAACATGCTGATTGCCGTGGTGGTGGCGTTCTTCATCTGCTGGGC

CCCGTTCCACGCTCAGCGCCTGATGGCCGTCTACGCAAAGGTGCCGACGCCGGCGCTCGAGATTGCCTTC

AACCTGCTCACGTACGTCTCTGGCGTCACGTACTACGTGAGCGCCACCATCAACCCCATCCTGTACAGCA

TCATGTCGCTCAAGTTCCGCCAGGCCTTCCGGGACACGCTCATGCGCTGCTGCGGCCGCCACCGCGCCAC

GCGCCATGAATGGAACTCGGCGGAGTGCTACGTCTCAAACCACCAGCTTCACACGACACCGTCGACGGTG

**TGA**ACAACACACCCTCGCCGCCAAGTACAGCGACCTGCTAACGTTTTCTCCCCTCAAGACTGTGCACTGT

GACTGTGGTTCCTGTGGGAATTACCTTCACGAGTATCTTAGCAGACAATATGGACGTCTTGCCACTTCGA

AGTGACAACGGCGAGTGTATTTTGCCTGCGAAAGCTTACTCTCTGTACCAAGACTGTAAAAAAAAGTGGC

TGTGGATTAACAAAAAAAAAAAAAAAAAAAAAA

Green =dsRNA PKR 762-913

Grey = dsRNA PKR 1485-1627

**RmPyr5’ dual luciferase construct (RmPyr_DualLuc-5’) 895 bp**

CGCGGTGGTGTTGTGTTCGTGGACGAGGTGCCTAAAGGACTGACCGGCAAGTTGGACGCCCGCAAGATCCGCGAGATTCTCATTAAGGCCAAGAAGGGCGGCAAGATCGCCGTGTAATTCTAGTTGTTTAAACGAGCTCGAGATCTGCAGCTGGTACCCGGGTCTAGAATTGCCCTTTTTCGCGCGGAGCTACGCGGACCTTTCAGGGACGTGCGTCAGCGGGCTTCGGGTGCTTTGATGGAGTGTCAGTCTATCTGTAACGTTAAAAGGCTCGAGTAATATTTTTTTGTTGGATCAATTTAAACACATGAAGAAATGATCCGAAGTGACTCTCTGTATTGTCCTGCGTTTTGTGATGACTACCAGCGAACTTTCGCTTGATTTGGAGCCCACTGCCCCTCCACTGATTCTTCGAACTCATCTGCGGATAATCTCTTGGTTCGGTGTTGACATTGCGTCCCGGTTGCACAGCGCTGGACGTGAAGAAACACGGCCGACAAAAGATCATGGACGAAAACTGTTGACAGTCACCTAGAAAAAAAGGAATTGTCTGCAGTTCACCTATAATGGCCTTAGTTTTTTCGATGACGGCTGGATTTGTGAGTGTACAACGGACAGCACCTTTCACAGTTACTTAAATCCGTCATTTACGGCCTGGGGACTTTCGACTGCGAGAAGA**TTCGAGCTGCAAAGAGTTCACAGTGCCCCTTCATCTCCTCTTTTCTACTATATCAAAACGCAGCGAGAGGAGCCTAAATGAG**ACGGCCACAATGAAACTGTCAAGCAGGTCGAGGCAGATCGGATCCCAATCTGAGGCCGGAGGAGC**ATG**ATCCCACCACAGCTGGCGTCCACCCTGGCGCACACGGACGCTGCGACCGACATGGCTTCCGCCGAA

Green =dsRNA PKR 762-913

**RmPyr3’ dual luciferase construct (RmPyr_DualLuc-3’) 1675 bp**

AGTTCTTAGTGCCCCTTCATCTCCTCTTTTCTGCTATATCAAAACGCAGCGAGAGGAGCCTCAACGAGTGCGTCAGCGGGCTTCGAACAAGCAGGCTGAGGCAGATCGGATCCCAATCTGAGACCGGAGGAGC**ATG**ATCCCACCACAGCCGGCGTCCACCCTGGCGCACACGGACGCTGCGACCGACATGGCTTCCGCCGACGAAGACGACACCGGTACGCTGGCCGACGATCCCCTGGTGACGCTGAACGCGTCAGCAGCCGCCGAAATGCTCCTCCTGGCATTGGGCCCCAAGCGCGACCCGCTAACCACGGTCATCCCAATGACGCTCATCTACTCTGTCCTGCTCGTCTCCGGAGTGGTCGGCAACGTCTGCACGTGCATCGTCATCGCGCGCAACCGGTACATGCACACGGCGACCAACTACTACCTCTTCAGCTTGGCCGTCTCCGATCTGCTCCTGCTGGTGCTCGGGCTGCCCCAAGAACTGTACCAACTCTGGCAGCGGCACCCGTACGTGTTCGGAGAGGCATTCTGCGTGCTCCGAGGCCTGACCTCGGAGACCTCGACCAACGCGTCCATACTGACCATCACCGCGTTCACGATCGAGCGCTACGTGGCGATCTGCCACCCGCTGCGGGCCCACACCATGTCCAAGTTGTCCCGGGCCGTCAAGTTTGTCGTCGCCATTTGGGTCCTGAGTGCCGTGTGTGCCGTCCCGCTCGCCGTCCAGTTCGGCATCGTCCACCAGACGCTGGACGGCACGACGGTGCTTCCGGAGACGGCCGCGTGCACGGTCAAGGACCCACTGGAACACGCCTTCGAACTGTCCACGTTCGTGTTCTTCTTGCTGCCCATGAGCGTCATTCTGGTGCTGTACGTTTGCATAGCGCTGCAGCTGAAGCGCTCCGACGCCCTCTCGCGCCAGGACGTGCACCACAAGTGTCCCGCCTCGAACAGCCCCAGCACCAGTGTCGTCAACGGGAAGGGCGACTCCTCGTCCACCGCGGGGCACAAAACAGAGTTCGGCTGTGGTGCAGCCGCTGCCCAGCAAGCTGCAGAGGGGCTGCCAGCTGAGGAAATCGGTTCGCGGAGGTGCGGCCGCGTCATCGTCCCGCAAGGCGGTCATCAACATGCTGATTGCCGTGGTGGTGGCGTTCTTCATCTGCTGGGCCCCGTTCCACGCTCAGCGCCTGATGGCCGTCTACGCAAAGGTGCCGACGCCGGCGCTCGAGATTGCCTTCAACCTGCTCACGTACGTCTCTGGCGTCACGTACTACGTGAGCGCCACCATCAACCCCATCCTGTACAGCATCATGTCGCTCAAGTTCCGCCAGGCCTTCCGGGACACGCTCATGCGCTGCTGCGGCCGCCACCGCGCCACGCGCCATGAATGGAACTCGGCGGAGTGCTACGTCTCAAACCACCAGCTTCACACGACACCGTCGACGGTG**TGA**ACAACACACCCTCGCCGCCAAGTACAGCGACCTGCTAACGTTTTCTCCCCCTCAAGACTGTGCACTGTGACTGTGGTTCCTGTGGGAATTACCTTCACGAGTATCTTAGCAGACAATGTGGACGTCTTGCCACTTCGAAGTGACAACGGCGAGTGTATTTTGCCTGCGAAAGCTTACTCTC

Grey = dsRNA PKR 1485-1627

**MAFFT alignment (DNASTAR Lasergene v11)**

1 50

*Rhimi-PKR*_(KP126932.1) ..................................................

RmPyr_DualLuc-5’ CGCGGTGGTGTTGTGTTCGTGGACGAGGTGCCTAAAGGACTGACCGGCAA

RmPyr_DualLuc-3' ..................................................

51 100

*Rhimi-PKR*_(KP126932.1) ..................................................

RmPyr_DualLuc-5’ GTTGGACGCCCGCAAGATCCGCGAGATTCTCATTAAGGCCAAGAAGGGCG

RmPyr_DualLuc-3' ..................................................

101 150

*Rhimi-PKR*_(KP126932.1) ..................................................

RmPyr_DualLuc-5’ GCAAGATCGCCGTGTAATTCTAGTTGTTTAAACGAGCTCGAGATCTGCAG

RmPyr_DualLuc-3' ..................................................

151 200

*Rhimi-PKR*_(KP126932.1) ..................................................

RmPyr_DualLuc-5’ CTGGTACCCGGGTCTAGAATTGCCCTTTTTCGCGCGGAGCTACGCGGACC

RmPyr_DualLuc-3' ..................................................

201 250

*Rhimi-PKR*_(KP126932.1) ..................................................

RmPyr_DualLuc-5’ TTTCAGGGACGTGCGTCAGCGGGCTTCGGGTGCTTTGATGGAGTGTCAGT

RmPyr_DualLuc-3' ..................................................

251 300

*Rhimi-PKR*_(KP126932.1) ..................................................

RmPyr_DualLuc-5’ CTATCTGTAACGTTAAAAGGCTCGAGTAATATTTTTTTGTTGGATCAATT

RmPyr_DualLuc-3' ..................................................

301 350

*Rhimi-PKR*_(KP126932.1) ..................................................

RmPyr_DualLuc-5’ TAAACACATGAAGAAATGATCCGAAGTGACTCTCTGTATTGTCCTGCGTT

RmPyr_DualLuc-3' ..................................................

351 400

*Rhimi-PKR*_(KP126932.1) ..................................................

RmPyr_DualLuc-5’ TTGTGATGACTACCAGCGAACTTTCGCTTGATTTGGAGCCCACTGCCCCT

RmPyr_DualLuc-3' ..................................................

401 450

*Rhimi-PKR*_(KP126932.1) ..................................................

RmPyr_DualLuc-5’ CCACTGATTCTTCGAACTCATCTGCGGATAATCTCTTGGTTCGGTGTTGA

RmPyr_DualLuc-3' ..................................................

451 500

*Rhimi-PKR*_(KP126932.1) ..................................................

RmPyr_DualLuc-5’ CATTGCGTCCCGGTTGCACAGCGCTGGACGTGAAGAAACACGGCCGACAA

RmPyr_DualLuc-3' ..................................................

501 550

*Rhimi-PKR*_(KP126932.1) ..................................................

RmPyr_DualLuc-5’ AAGATCATGGACGAAAACTGTTGACAGTCACCTAGAAAAAAAGGAATTGT

RmPyr_DualLuc-3' ..................................................

551 600

*Rhimi-PKR*_(KP126932.1) ..................................................

RmPyr_DualLuc-5’ CTGCAGTTCACCTATAATGGCCTTAGTTTTTTCGATGACGGCTGGATTTG

RmPyr_DualLuc-3' ..................................................

601 650

*Rhimi-PKR*_(KP126932.1) ..................................................

RmPyr_DualLuc-5’ TGAGTGTACAACGGACAGCACCTTTCACAGTTACTTAAATCCGTCATTTA

RmPyr_DualLuc-3' ..................................................

651 700

*Rhimi-PKR*_(KP126932.1) .............................TTCGAGCCGGAAAGAGTTCTT

RmPyr_DualLuc-5’ CGGCCTGGGGACTTTCGACTGCGAGAAGATTCGAGCTGCAAAGAGTTCAC

RmPyr_DualLuc-3' ...........................................AGTTCTT

701 750

*Rhimi-PKR*_(KP126932.1) AGTGCCCCTTCATCTCCTCTTTTCTGCTATATCAAAACGCAGCGAGAGGA

RmPyr_DualLuc-5’ AGTGCCCCTTCATCTCCTCTTTTCTACTATATCAAAACGCAGCGAGAGGA

RmPyr_DualLuc-3' AGTGCCCCTTCATCTCCTCTTTTCTGCTATATCAAAACGCAGCGAGAGGA

751 800

*Rhimi-PKR*_(KP126932.1) GCCTCAACGAGACGGCCACAATGGAACTGTCAAGCAGGCTGAGGCAGATC

RmPyr_DualLuc-5’ GCCTAAATGAGACGGCCACAATGAAACTGTCAAGCAGGTCGAGGCAGATC

RmPyr_DualLuc-3' GCCTCAACGAGTGCGTCAGCGGGCTTCGAACAAGCAGGCTGAGGCAGATC

801 850

*Rhimi-PKR*_(KP126932.1) GGATCCCAATCTGAGACCGGAGGAGC**ATG**ATCCCACCACAGCCGGCGTCC

RmPyr_DualLuc-5’ GGATCCCAATCTGAGGCCGGAGGAGCATGATCCCACCACAGCTGGCGTCC

RmPyr_DualLuc-3' GGATCCCAATCTGAGACCGGAGGAGCATGATCCCACCACAGCCGGCGTCC

851 900

*Rhimi-PKR*_(KP126932.1) ACCCTGGCGCACACGGACGCTGCGACCGACATGGCTTCCGCCGACGAAGA

RmPyr_DualLuc-5’ ACCCTGGCGCACACGGACGCTGCGACCGACATGGCTTCCGCCGAA.....

RmPyr_DualLuc-3' ACCCTGGCGCACACGGACGCTGCGACCGACATGGCTTCCGCCGACGAAGA

901 950

*Rhimi-PKR*_(KP126932.1) CGACACCGGTACGCAGGCCGACGATCCCCTGGTGACGCTGAACGCGTCAG

RmPyr_DualLuc-5’ ..................................................

RmPyr_DualLuc-3' CGACACCGGTACGCTGGCCGACGATCCCCTGGTGACGCTGAACGCGTCAG

951 1000

*Rhimi-PKR*_(KP126932.1) CAGCCGCCGAAATGCTCCTCCTGGCATTGGGCCCCAAGCGCGACCCGCTA

RmPyr_DualLuc-5’ ..................................................

RmPyr_DualLuc-3' CAGCCGCCGAAATGCTCCTCCTGGCATTGGGCCCCAAGCGCGACCCGCTA

1001 1050

*Rhimi-PKR*_(KP126932.1) ACCACGGTCATCCCAATGACGCTCATCTACTCTGTCCTGCTCGTCTCCGG

RmPyr_DualLuc-5’ ..................................................

RmPyr_DualLuc-3' ACCACGGTCATCCCAATGACGCTCATCTACTCTGTCCTGCTCGTCTCCGG

1051 1100

*Rhimi-PKR*_(KP126932.1) AGTGGTCGGCAACGTCTGCACGTGCATCGTCATCGCGCACAACCGGTACA

RmPyr_DualLuc-5’ ..................................................

RmPyr_DualLuc-3' AGTGGTCGGCAACGTCTGCACGTGCATCGTCATCGCGCGCAACCGGTAC.

1101 1150

*Rhimi-PKR*_(KP126932.1) TGATGCACACGGCGACCAACTACTACCTCTTCAGCTTGGCCGTCTCCGAT

RmPyr_DualLuc-5’ ..................................................

RmPyr_DualLuc-3' ..ATGCACACGGCGACCAACTACTACCTCTTCAGCTTGGCCGTCTCCGAT

1151 1200

*Rhimi-PKR*_(KP126932.1) CTGCTCCTGCTGGTGCTCGGGCTGCCCCAAGAACTGTACCAACTCTGGCA

RmPyr_DualLuc-5’ ..................................................

RmPyr_DualLuc-3' CTGCTCCTGCTGGTGCTCGGGCTGCCCCAAGAACTGTACCAACTCTGGCA

1201 1250

*Rhimi-PKR*_(KP126932.1) GCGGCACCCGTACGTGTTCGGAGAGGCATTCTGCGTGCTCCGGGGCCTGA

RmPyr_DualLuc-5’ ..................................................

RmPyr_DualLuc-3' GCGGCACCCGTACGTGTTCGGAGAGGCATTCTGCGTGCTCCGAGGCCTGA

1251 1300

*Rhimi-PKR*_(KP126932.1) CCTCGGAGACCTCAACCAACGCGTCCATACTGACCATCACCGCGTTCACG

RmPyr_DualLuc-5’ ..................................................

RmPyr_DualLuc-3' CCTCGGAGACCTCGACCAACGCGTCCATACTGACCATCACCGCGTTCACG

1301 1350

*Rhimi-PKR*_(KP126932.1) ATCGAGCGCTACGTGGCGATCTGCCACCCGCTGCGGGCCCACACCATGTC

RmPyr_DualLuc-5’ ..................................................

RmPyr_DualLuc-3' ATCGAGCGCTACGTGGCGATCTGCCACCCGCTGCGGGCCCACACCATGTC

1351 1400

*Rhimi-PKR*_(KP126932.1) CAAGTTGTCCCGGGCCGTCAAATTTGTCGTCGCCATTTGGGTCCTAAGCG

RmPyr_DualLuc-5’ ..................................................

RmPyr_DualLuc-3' CAAGTTGTCCCGGGCCGTCAAGTTTGTCGTCGCCATTTGGGTCCTGAGTG

1401 1450

*Rhimi-PKR*_(KP126932.1) CCGTGTGTGCCGTCCCGCTCGCCGTCCAGTTCGGCATCGTCCACCAGACG

RmPyr_DualLuc-5’ ..................................................

RmPyr_DualLuc-3' CCGTGTGTGCCGTCCCGCTCGCCGTCCAGTTCGGCATCGTCCACCAGACG

1451 1500

*Rhimi-PKR*_(KP126932.1) CTGGACGGCACGACGGTGCTTCCGGAGACGGCCGCGTGCACGGTCAAGGA

RmPyr_DualLuc-5’ ..................................................

RmPyr_DualLuc-3' CTGGACGGCACGACGGTGCTTCCGGAGACGGCCGCGTGCACGGTCAAGGA

1501 1550

*Rhimi-PKR*_(KP126932.1) CCCGCTGGAACACGCCTTCGAACTGTCCACGTTCGTGTTCTTCTTGCTGC

RmPyr_DualLuc-5’ ..................................................

RmPyr_DualLuc-3' CCCACTGGAACACGCCTTCGAACTGTCCACGTTCGTGTTCTTCTTGCTGC

1551 1600

*Rhimi-PKR*_(KP126932.1) CCATGAGCGTCATTCTGGTGCTGTACGTTTGCATAGCGCTGCAGCTGAAG

RmPyr_DualLuc-5’ ..................................................

RmPyr_DualLuc-3' CCATGAGCGTCATTCTGGTGCTGTACGTTTGCATAGCGCTGCAGCTGAAG

1601 1650

*Rhimi-PKR*_(KP126932.1) CGCTCCGACGCCCTGTCGCGCCGGGACGTGCACCACAAGTGTCCCGCCTC

RmPyr_DualLuc-5’ ..................................................

RmPyr_DualLuc-3' CGCTCCGACGCCCTCTCGCGCCAGGACGTGCACCACAAGTGTCCCGCCTC

1651 1700

*Rhimi-PKR*_(KP126932.1) GAACAGCCCCAGCACCAGTGTCGTCAACGGGAAGGGCGACTCCTCGTCCA

RmPyr_DualLuc-5’ ..................................................

RmPyr_DualLuc-3' GAACAGCCCCAGCACCAGTGTCGTCAACGGGAAGGGCGACTCCTCGTCCA

1701 1750

*Rhimi-PKR*_(KP126932.1) CCGTGGGGCAC.AAACAGAGTTCGGCTGTGGTGCAGCCGCTGCCCAGCAA

RmPyr_DualLuc-5’ ..................................................

RmPyr_DualLuc-3' CCGCGGGGCACAAAACAGAGTTCGGCTGTGGTGCAGCCGCTGCCCAGCAA

1751 1800

*Rhimi-PKR*_(KP126932.1) GCTGCAGAGGGGCTGCCAGCTGAGGAAATCGGTTCGCGGAGGTGCGGCCG

RmPyr_DualLuc-5’ ..................................................

RmPyr_DualLuc-3' GCTGCAGAGGGGCTGCCAGCTGAGGAAATCGGTTCGCGGAGGTGCGGCCG

1801 1850

*Rhimi-PKR*_(KP126932.1) CGTCATCGTCCCGCAAGGCGGTCATCAACATGCTGATTGCCGTGGTGGTG

RmPyr_DualLuc-5’ ..................................................

RmPyr_DualLuc-3' CGTCATCGTCCCGCAAGGCGGTCATCAACATGCTGATTGCCGTGGTGGTG

1851 1900

*Rhimi-PKR*_(KP126932.1) GCGTTCTTCATCTGCTGGGCCCCGTTCCACGCTCAGCGCCTGATGGCCGT

RmPyr_DualLuc-5’ ..................................................

RmPyr_DualLuc-3' GCGTTCTTCATCTGCTGGGCCCCGTTCCACGCTCAGCGCCTGATGGCCGT

1901 1950

*Rhimi-PKR*_(KP126932.1) CTACGCAAAGGTGCCGACGCCGGCGCTCGAGATTGCCTTCAACCTGCTCA

RmPyr_DualLuc-5’ ..................................................

RmPyr_DualLuc-3' CTACGCAAAGGTGCCGACGCCGGCGCTCGAGATTGCCTTCAACCTGCTCA

1951 2000

*Rhimi-PKR*_(KP126932.1) CGTACGTCTCTGGCGTCACGTACTACGTGAGCGCCACCATCAACCCCATC

RmPyr_DualLuc-5’ ..................................................

RmPyr_DualLuc-3' CGTACGTCTCTGGCGTCACGTACTACGTGAGCGCCACCATCAACCCCATC

2001 2050

*Rhimi-PKR*_(KP126932.1) CTGTACAGCATCATGTCGCTCAAGTTCCGCCAGGCCTTCCGGGACACGCT

RmPyr_DualLuc-5’ ..................................................

RmPyr_DualLuc-3' CTGTACAGCATCATGTCGCTCAAGTTCCGCCAGGCCTTCCGGGACACGCT

2051 2100

*Rhimi-PKR*_(KP126932.1) CATGCGCTGCTGCGGCCGCCACCGCGCCACGCGCCATGAATGGAACTCGG

RmPyr_DualLuc-5’ ..................................................

RmPyr_DualLuc-3' CATGCGCTGCTGCGGCCGCCACCGCGCCACGCGCCATGAATGGAACTCGG

2101 2150

*Rhimi-PKR*_(KP126932.1) CGGAGTGCTACGTCTCAAACCACCAGCTTCACACGACACCGTCGACGGTG

RmPyr_DualLuc-5’ ..................................................

RmPyr_DualLuc-3' CGGAGTGCTACGTCTCAAACCACCAGCTTCACACGACACCGTCGACGGTG

2151 2200

*Rhimi-PKR*_(KP126932.1) **TGA**ACAACACACCCTCGCCGCCAAGTACAGCGACCTGCTAACGTTTTCT.

RmPyr_DualLuc-5’ ..................................................

RmPyr_DualLuc-3' TGAACAACACACCCTCGCCGCCAAGTACAGCGACCTGCTAACGTTTTCTC

2201 2250

*Rhimi-PKR*_(KP126932.1) CCCCTCAAGACTGTGCACTGTGACTGTGGTTCCTGTGGGAATTACCTTCA

RmPyr_DualLuc-5’ ..................................................

RmPyr_DualLuc-3' CCCCTCAAGACTGTGCACTGTGACTGTGGTTCCTGTGGGAATTACCTTCA

2251 2300

*Rhimi-PKR*_(KP126932.1) CGAGTATCTTAGCAGACAATATGGACGTCTTGCCACTTCGAAGTGACAAC

RmPyr_DualLuc-5’ ..................................................

RmPyr_DualLuc-3' CGAGTATCTTAGCAGACAATGTGGACGTCTTGCCACTTCGAAGTGACAAC

2301 2350

*Rhimi-PKR*_(KP126932.1) GGCGAGTGTATTTTGCCTGCGAAAGCTTACTCTCTGTACCAAGACTGTAA

RmPyr_DualLuc-5’ ..................................................

RmPyr_DualLuc-3' GGCGAGTGTATTTTGCCTGCGAAAGCTTACTCTC----------------

**Cholinesterase-like (XM_037420972.1) fragment sequence (dsfsg), used as dsRNA negative control in the RNAi in vitro assay) – 357 bp**

CTTTCCCGGATAGTCGAATCCAAACGGAGGAGCTCGAAGGCATCAGCGAAGAAGAGCTCAAGAAAGTTCTGCGTGTCATGTCCCTCGCCTGGATTCCGGATAAGTTCGCGTCTACGCTGGAGCACTATGCTAGCGCAGCCACTCCGGGTGACAAGCGACTTCTGAGGGAATTGCATATGGAGTATATAACTGACGCACAGTTCATTTGTCCCAGCAAGTTCTTTGCCCAAGACTACTCTGAAATGGGTAACTCTGTGTACTTTTCCGTGCTGGGCTACAGGTCGGCGAAGTTTCCGTTTCCAAAGTGGACTCGCGTACCCCACACCTCTGACATCGTCTACTACTTCGGCGTTCCTC
