# Supplementary material for: Pyrokinin receptor silencing in females of the southern cattle tick Rhipicephalus (Boophilus) microplus is associated with a reproductive fitness cost
Source: Parasit Vectors. 2022 Jul 11;15:252. doi: 10.1186/s13071-022-05349-w (PMC9272880; doi:10.1186/s13071-022-05349-w)
Supplement: Supplementary file 2 — Additional file 2: Figure S2. NCBI-BLASTn searches to check for possible off-target effects of the PKR dsRNA sequences. [file 13071_2022_5349_MOESM2_ESM.docx]

Primer off-target BLASTn Search for all dsRNA constructs – NCBI (*Ripicephalus microplus* genome)

Query #1: **ds762-913** Query ID: lcl|Query_508334 Length: 131

Alignments:

1)*Rhimi*-PKR

>Rhipicephalus microplus isolate Rmic-2018 chromosome 11, ASM1333972v1

Sequence ID: NC_051175.1 Length: 126224405

Range 1: 117170980 to 117171110

Score:219 bits (242), Expect:2e-55,

Identities:127/131(97%), Gaps:0/131(0%), Strand: Plus/Minus

Query 1 ACGGCCACAATGAAACTGTCAAGCAGGTCGAGGCAGATCGGATCCCAATCTGAGGCCGGA 60

||||||||||||||||||||||||||| |||||||||||| |||||||||||||||||||

Sbjct 117171110 ACGGCCACAATGAAACTGTCAAGCAGGCCGAGGCAGATCGCATCCCAATCTGAGGCCGGA 117171051

Query 61 GGAGCATGATCCCACCACAGCTGGCGTCCACCCTGGCGCACACGGACGCTGCGACCGACA 120

||||||||||||||||||||| |||||||||||||||||| |||||||||||||||||||

Sbjct 117171050 GGAGCATGATCCCACCACAGCCGGCGTCCACCCTGGCGCATACGGACGCTGCGACCGACA 117170991

Query 121 TGGCTTCCGCC 131

|||||||||||

Sbjct 117170990 TGGCTTCCGCC 117170980

Range 2: 30989667 to 30989685

Score:35.6 bits(38), Expect:5.1,

Identities:19/19(100%), Gaps:0/19(0%), Strand: Plus/Minus

Query 13 AAACTGTCAAGCAGGTCGA 31

|||||||||||||||||||

Sbjct 30989685 AAACTGTCAAGCAGGTCGA 30989667

2) Off-target

>Rhipicephalus microplus isolate Rmic-2018 chromosome 3, ASM1333972v1

Sequence ID: NC_051167.1 Length: 238537928

Range 1: 31630508 to 31630533

Score:40.1 bits (43), Expect:0.42,

Identities:26/28(93%), Gaps:2/28(7%), Strand: Plus/Minus

Query 74 ACCACAGCTGGCGTCCACCCTGGCGCAC 101

||||||||||||||||| |||||||||

Sbjct 31630533 ACCACAGCTGGCGTCCA--CTGGCGCAC 31630508

Range 2: 186635656 to 186635679

Score:36.5 bits (39), Expect:5.1,

Identities:24/26(92%), Gaps:2/26(7%), Strand: Plus/Minus

Query 74 ACCACAGCTGGCGTCCACCCTGGCGC 99

||||||||||||||||| |||||||

Sbjct 186635679 ACCACAGCTGGCGTCCA--CTGGCGC 186635656

3) Off-target

>Rhipicephalus microplus isolate Rmic-2018 unplaced genomic scaffold, ASM1333972v1 Seq7897

Sequence ID: NW_023613883.1 Length: 2626766

Range 1: 1155792 to 1155816

Score:38.3 bits (41), Expect:1.5,

Identities:25/27(93%), Gaps:2/27(7%), Strand: Plus/Plus

Query 75 CCACAGCTGGCGTCCACCCTGGCGCAC 101

|||||||||||||| |||||||||||

Sbjct 1155792 CCACAGCTGGCGTC--CCCTGGCGCAC 1155816

4) Off-target

>Rhipicephalus microplus isolate Rmic-2018 chromosome 1, ASM1333972v1

Sequence ID: NC_051165.1 Length: 325144201

Range 1: 13469755 to 13469780

Score:35.6 bits (38), Expect:5.1,

Identities:25/28(89%), Gaps:2/28(7%), Strand: Plus/Plus

Query 75 CCACAGCTGGCGTCCACCCTGGCGCACA 102

|||||||||||||||| ||||||| ||

Sbjct 13469755 CCACAGCTGGCGTCCA--CTGGCGCGCA 13469780

5) Off-target

>Rhipicephalus microplus isolate Rmic-2018 chromosome 2, ASM1333972v1

Sequence ID: NC_051166.1 Length: 202141382

Range 1: 81973247 to 81973270

Score:35.6 bits (38), Expect:5.1,

Identities:22/24(92%), Gaps:0/24(0%), Strand: Plus/Plus

Query 108 GCTGCGACCGACATGGCTTCCGCC 131

||||||| || |||||||||||||

Sbjct 81973247 GCTGCGAGCGCCATGGCTTCCGCC 81973270

6) Off-target

>Rhipicephalus microplus isolate Rmic-2018 chromosome 8, ASM1333972v1

Sequence ID: NC_051172.1 Length: 170387423

Range 1: 54482141 to 54482164

Score:35.6 bits (38), Expect:5.1,

Identities:22/24(92%), Gaps:0/24(0%), Strand: Plus/Plus

Query 58 GGAGGAGCATGATCCCACCACAGC 81

||||||||| || |||||||||||

Sbjct 54482141 GGAGGAGCAAGAACCCACCACAGC 54482164

7)Off-target

>Rhipicephalus microplus isolate Rmic-2018 unplaced genomic scaffold, ASM1333972v1 Seq7930

Sequence ID: NW_023613889.1 Length: 3309063

Range 1: 2177652 to 2177677

Score:35.6 bits (38), Expect:5.1,

Identities:25/28(89%), Gaps:2/28(7%), Strand: Plus/Minus

Query 74 ACCACAGCTGGCGTCCACCCTGGCGCAC 101

||||||||||||||| | |||||||||

Sbjct 2177677 ACCACAGCTGGCGTC--CGCTGGCGCAC 2177652

Query #2: **ds1485-1627** Query ID: lcl|Query_508335 Length: 127

Alignments:

1)*Rhimi*-PKR

>Rhipicephalus microplus isolate Rmic-2018 chromosome 11, ASM1333972v1

Sequence ID: NC_051175.1 Length: 126224405

Range 1: 117022967 to 117023093

Score:225 bits (249), Expect:5e-57,

Identities:126/127(99%), Gaps:0/127(0%), Strand: Plus/Minus

Query 1 CGACCTGCTAACGTTTTCTCCCCCTCAAGACTGTGCACTGTGACTGTGGTTCCTGTGGGA 60

||||||||||||||||||||||||||||||||||||||||||||||||||||||||||||

Sbjct 117023093 CGACCTGCTAACGTTTTCTCCCCCTCAAGACTGTGCACTGTGACTGTGGTTCCTGTGGGA 117023034

Query 61 ATTACCTTCACGAGTATCTTAGCAGACAATGTGGACGTCTTGCCACTTCGAAGTGACAAC 120

|||||||||||||||||||||||||||||| |||||||||||||||||||||||||||||

Sbjct 117023033 ATTACCTTCACGAGTATCTTAGCAGACAATATGGACGTCTTGCCACTTCGAAGTGACAAC 117022974

Query 121 GGCGAGT 127

|||||||

Sbjct 117022973 GGCGAGT 117022967

2) Off-target

>Rhipicephalus microplus isolate Rmic-2018 chromosome 2, ASM1333972v1

Sequence ID: NC_051166.1 Length: 202141382

Range 1: 179471429 to 179471459

Score:37.4 bits (40), Expect:1.4,

Identities:28/32(88%), Gaps:1/32(3%), Strand: Plus/Plus

Query 81 AGCAGACAATGTGGACGTCTTGCCACTTCGAA 112

|||| ||||| | ||||||||||||| |||||

Sbjct 179471429 AGCA-ACAATCTAGACGTCTTGCCACCTCGAA 179471459

3) Off-target

>Rhipicephalus microplus isolate Rmic-2018 chromosome 7, ASM1333972v1

Sequence ID: NC_051171.1 Length: 175432524

Range 1: 31836851 to 31836879

Score:37.4 bits (40), Expect:1.4,

Identities:26/29(90%), Gaps:2/29(6%), Strand: Plus/Minus

Query 87 CAATGTGGACGTCTTGCCAC--TTCGAAG 113

|| ||||||||||||||||| |||||||

Sbjct 31836879 CATTGTGGACGTCTTGCCACGGTTCGAAG 31836851

4) Off-target

>Rhipicephalus microplus isolate Rmic-2018 chromosome 4, ASM1333972v1

Sequence ID: NC_051168.1 Length: 218457896

Range 1: 77427983 to 77428004

Score:36.5 bits (39), Expect:4.9,

Identities:21/22(95%), Gaps:0/22(0%), Strand: Plus/Minus

Query 24 CTCAAGACTGTGCACTGTGACT 45

|||| |||||||||||||||||

Sbjct 77428004 CTCACGACTGTGCACTGTGACT 77427983

5) Off-target

>Rhipicephalus microplus isolate Rmic-2018 chromosome 8, ASM1333972v1

Sequence ID: NC_051172.1 Length: 170387423

Range 1: 18530783 to 18530804

Score:36.5 bits (39), Expect:4.9,

Identities:21/22(95%), Gaps:0/22(0%), Strand: Plus/Plus

Query 71 CGAGTATCTTAGCAGACAATGT 92

|||||||||||| |||||||||

Sbjct 18530783 CGAGTATCTTAGAAGACAATGT 18530804

6) Off-target

>Rhipicephalus microplus isolate Rmic-2018 chromosome 9, ASM1333972v1

Sequence ID: NC_051173.1 Length: 155099419

Range 1: 25531512 to 25531535

Score:35.6 bits (38), Expect:4.9,

Identities:22/24(92%), Gaps:0/24(0%), Strand: Plus/Plus

Query 32 TGTGCACTGTGACTGTGGTTCCTG 55

||||||||||||| ||| ||||||

Sbjct 25531512 TGTGCACTGTGACAGTGTTTCCTG 25531535

7) Off-target

>Rhipicephalus microplus isolate Rmic-2018 unplaced genomic scaffold, ASM1333972v1 Seq521

Sequence ID: NW_023611608.1 Length: 38418

Range 1: 11842 to 11865

Score:35.6 bits (38), Expect:4.9,

Identities:22/24(92%), Gaps:0/24(0%), Strand: Plus/Plus

Query 29 GACTGTGCACTGTGACTGTGGTTC 52

||||||||||||||| |||| |||

Sbjct 11842 GACTGTGCACTGTGAATGTGATTC 11865

8) Off-target

>Rhipicephalus microplus isolate Rmic-2018 unplaced genomic scaffold, ASM1333972v1 Seq5551

Sequence ID: NW_023611980.1 Length: 139416

Range 1: 119709 to 119740

Score:35.6 bits (38), Expect:4.9,

Identities:29/33(88%), Gaps:2/33(6%), Strand: Plus/Plus

Query 26 CAAGACTGT-GCACTGTGACTGTGGTTCCTGTG 57

||| ||||| ||||||||||||| || ||||||

Sbjct 119709 CAACACTGTCGCACTGTGACTGT-GTGCCTGTG 119740
