# Supplementary figures and images for: Pyrokinin receptor silencing in females of the southern cattle tick Rhipicephalus (Boophilus) microplus is associated with a reproductive fitness cost
Source: Parasit Vectors. 2022 Jul 11;15:252. doi: 10.1186/s13071-022-05349-w (PMC9272880; doi:10.1186/s13071-022-05349-w)

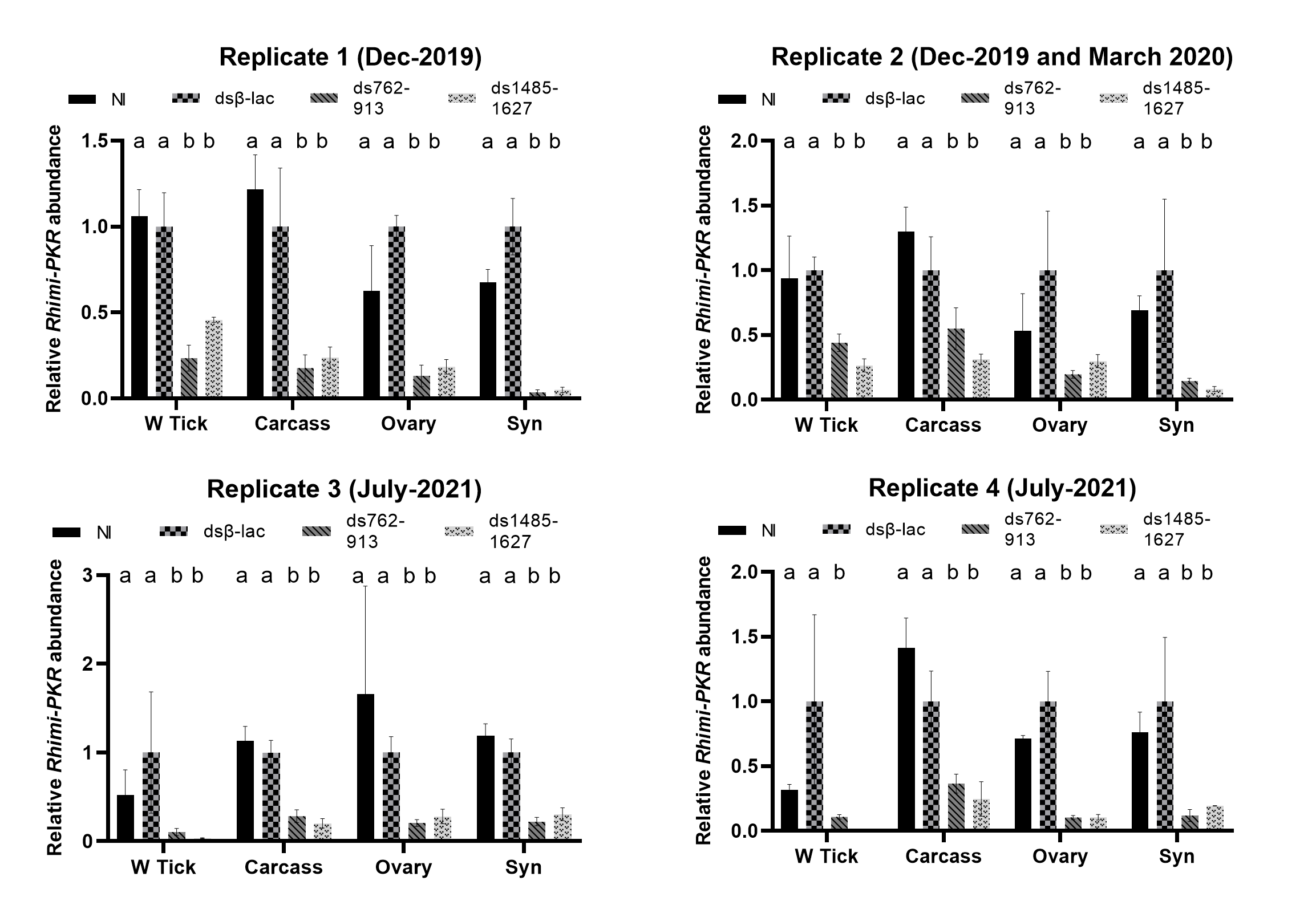

Supplement: Supplementary file 4 — Additional file 4: Figure S4. Results of qRT-PCR evaluating RNAi silencing of Rhimi-PKR shown for the independent replicates. A one-way ANOVA followed by a Tukey’s multiple comparisons test was used for the statistical analysis. Different lowercase letters indicate significant difference (P < 0.05). Abbreviations: NI, Non-injected; β-lac, beta-lactamase dsRNA-injected ticks (negative controls); ds762-913 and ds1485-1627, Rhimi-PKR dsRNA-injected ticks; W tick, whole tick; Syn, synganglion. [file 13071_2022_5349_MOESM4_ESM.tif]

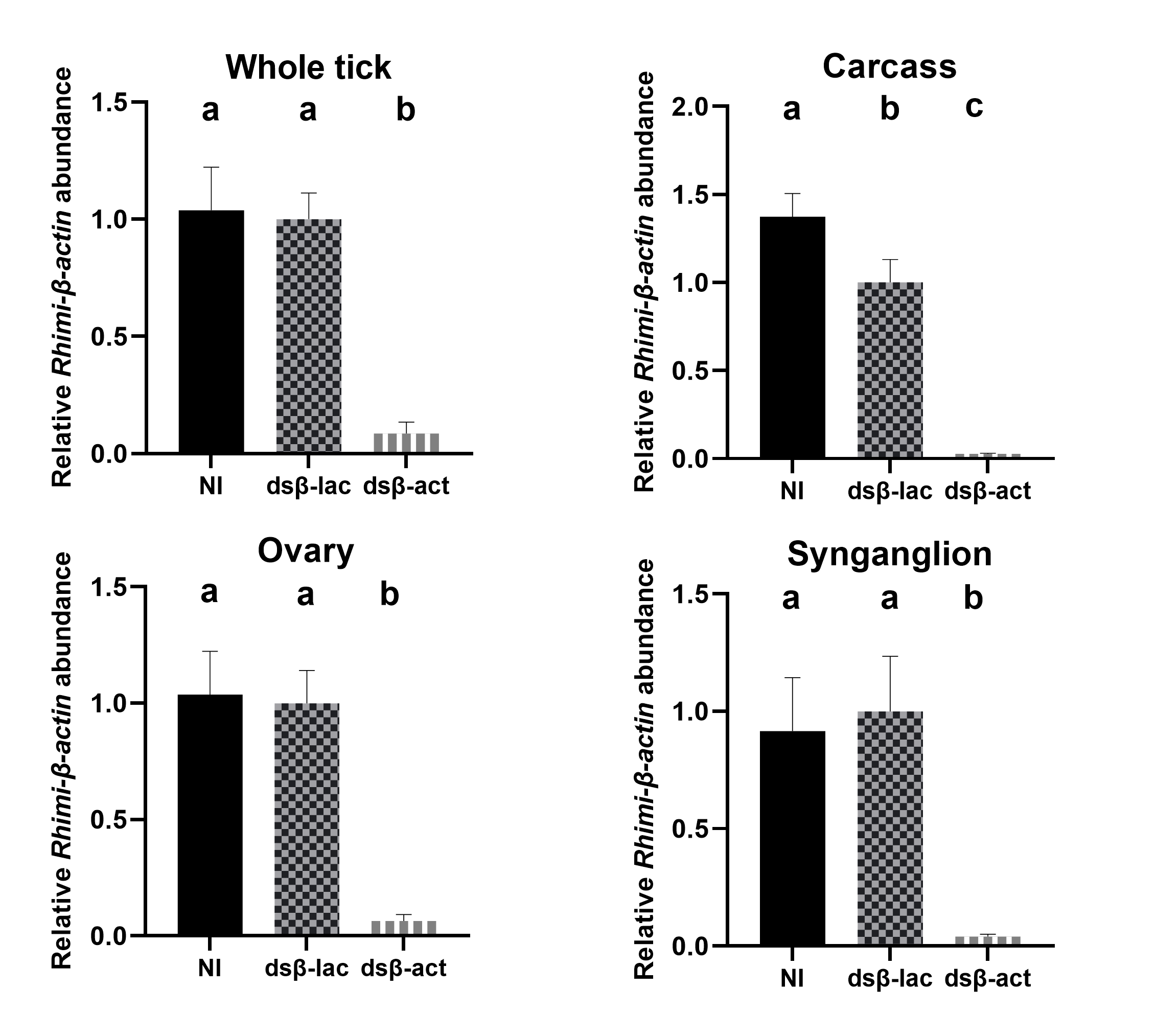

Supplement: Supplementary file 5 — Additional file 5: Figure S5. Verification of Rhimi-ACTB silencing as suitable positive control by qRT-PCR, for all replicates combined. A one-way ANOVA followed by a Tukey’s multiple comparisons test was used for the statistical analysis. Different lowercase letters in the figure indicate significant difference (P < 0.05). Abbreviations: NI, Non-injected; β-lac, beta-lactamase dsRNA-injected ticks (negative controls); dsβ-act, Rhimi-ACTB, dsRNA-injected ticks (positive control). [file 13071_2022_5349_MOESM5_ESM.tif]
